# Transcription factors containing both C_2_H_2_ and homeobox domains play different roles in *Verticillium dahliae*

**DOI:** 10.1128/msphere.00409-24

**Published:** 2024-08-27

**Authors:** Chen Tang, Haifeng Wang, Xianjiang Jin, Wenwen Li, Yonglin Wang

**Affiliations:** 1 State Key Laboratory of Efficient Production of Forest Resources, Beijing Key Laboratory for Forest Pest Control, College of Forestry, Beijing Forestry University, Beijing, China; University of Georgia, Athens, Georgia, USA

**Keywords:** transcription factor, C_2_H_2_-homeobox domain, microsclerotia, *Verticillium dahliae*

## Abstract

*Verticillium dahliae* causes Verticillium wilt in more than 200 plant species worldwide. As a soilborne fungus, it forms melanized microsclerotia and colonizes the xylem of host plants. Our previous study revealed a subfamily of C_2_H_2_-homeobox transcription factors in *V. dahliae*, but their biological roles remain unknown. In this study, we systematically characterized the functions of seven C_2_H_2_-homeobox transcription factors in *V. dahliae*. Deletion of *VdChtf3* and *VdChtf6* significantly decreased the production of melanized microsclerotia, and knockout of *VdChtf1* and *VdChtf4* enhanced virulence. Loss of *VdChtf2* and *VdChtf6* increased conidium production, whereas loss of *VdChtf5* and *VdChtf7* did not affect growth, conidiation, microsclerotial formation, or virulence. Further research showed that VdChtf3 activated the expression of genes encoding pectic enzymes to participate in microsclerotial formation. In addition, VdChtf4 reduced the expression of *VdSOD1* to disturb the scavenging of superoxide radicals but induced the expression of genes related to cell wall synthesis to maintain cell wall integrity. These findings highlight the diverse roles of different members of the C_2_H_2_-homeobox gene family in *V. dahliae*.

**IMPORTANCE:**

*Verticillium dahliae* is a soilborne fungus that causes plant wilt and can infect a variety of economic crops and woody trees. The molecular basis of microsclerotial formation and infection by this fungus remains to be further studied. In this study, we analyzed the functions of seven C2H2-homobox transcription factors. Notably, VdChtf3 and VdChtf4 exhibited the most severe defects, affecting phenotypes associated with critical developmental stages in the *V. dahliae* disease cycle. Our results indicate that VdChtf3 is a potential specific regulator of microsclerotial formation, modulating the expression of pectinase-encoding genes. This finding could contribute to a better understanding of microsclerotial development in *V. dahliae*. Moreover, VdChtf4 was associated with cell wall integrity, reactive oxygen species (ROS) stress resistance, and increased virulence. These discoveries shed light on the biological significance of C2H2-homeobox transcription factors in *V. dahliae*'s adaptation to the environment and infection of host plants.

## INTRODUCTION

*Verticillium dahliae*, a soilborne fungal pathogen that causes Verticillium wilt and even plant death, poses a significant threat to a wide range of plant species, including economically important crops such as cotton, tomato, and lettuce and ornamental plants such as smoke tree (1–3). This pathogen is difficult to control because of its broad host range and the persistence of its microsclerotia in soil (1, 2). Microsclerotia consist of compact masses of thick-walled, pigmented cells formed by budding from swollen, septate hyphae, serving as the main dormant survival structures as well as a new source of the primary inoculum in the disease cycle of *V. dahliae* (4, 5). Microsclerotia are highly resistant to adverse poor environmental conditions such as high temperatures and ultraviolet light (6, 7), allowing them to survive for more than 14 years in the soil (8, 9). Upon sensing the rhizospheric exudates of the host plant, the microsclerotia germinate and form hyphopodia and penetration pegs to infect the host from the root (2, 10). Therefore, understanding the molecular mechanisms involved in microsclerotial formation and infection could provide new strategies for the management of *V. dahliae*.

Transcription factors are proteins that bind to specific DNA sequences to regulate gene expression. Transcriptional regulation by transcription factors is an important regulatory mechanism in phytopathogenic fungi during development and infection. In *V. dahliae*, transcription factors account for more than 5% of all genes (11), and only a small fraction of them have been functionally characterized. Cys_2_His_2_ (C_2_H_2_) zinc finger proteins are among the main transcription factors in *V. dahliae*, some of which regulate microsclerotial formation and virulence. For example, the loss of *VdZFP1* and *VdZFP2* reduced melanized microsclerotia development (12), while the Δ*VdCf2* mutant increased melanin accumulation and impaired growth and virulence (13). Deletion of *VdCrz1* affects microsclerotial formation and virulence and increases sensitivity to high concentrations of Ca^2+^ and cell wall stress (14). VdMsn2 notably impacts hyphal growth and septation, microsclerotial formation, and virulence (15). Homeobox transcription factors are generally considered to be involved in morphogenesis and pathogenicity in fungal pathogens (16). In *V. dahliae*, the homeobox transcription factors vph1 and vhb1 are critical for virulence (17). However, the functions of other homeobox transcription factors in *V. dahliae* are poorly understood.

Only few transcription factors contain more than one type of DNA-binding domain (18). For instance, C_2_H_2_-homeobox transcription factors contain both a C_2_H_2_ zinc finger domain and a homeobox domain. C_2_H_2_-homeobox transcription factors do not exist in *Saccharomyces cerevisiae* (19), and *Magnaporthe oryzae* contains only one C_2_H_2_-homeobox transcription factor, MST12*,* which regulates cellular functions associated with appressorium maturation (20, 21). The C_2_H_2_-homeobox gene *SteA* regulates the secretion of cell wall-degrading enzymes to maintain cell wall integrity in *Aspergillus oryzae* (22). Previously, we identified seven C_2_H_2_-homeobox transcription factors in *V. dahliae* based on genomics studies, in addition to Ste12 (19). Vph1, the protein homolog of Ste12, contains both a C_2_H_2_ domain and a homeobox domain, affecting hyphal growth and virulence of *V. dahliae* (17). However, the biological functions of other seven C_2_H_2_-homeobox transcription factors remain to be further identified.

In this study, to further understand the role of these seven C_2_H_2_-homeobox transcription factors in *V. dahliae*, we generated all the mutant strains. The deletion of *VdChtf2* and *VdChtf6* led to conidium overproduction; the deletion of both *VdChtf3* and *VdChtf6* decreased microsclerotial formation; and the deletion of *VdChtf4* led to an increase in virulence and resistance to ROS. Considering the significant impact of VdChtf3 on microsclerotial formation, we further conducted RNA-seq analysis and found that VdChtf3 may regulate microsclerotial formation by modulating the expression of pectinase-encoding genes. We also found that the Δ*VdChtf4* strain exhibited enhanced resistance to hydrogen peroxide stress, which might be attributed to the upregulated expression of *VdSOD1*. In addition, VdChtf4 positively regulates genes related to cell wall synthesis and is involved in maintaining cell wall integrity. These results suggest that C_2_H_2_-homeobox genes play different roles in *V. dahliae* and improve our understanding of the development and pathogenesis of *V. dahliae*.

## RESULTS

### Phenotypes of the seven C_2_H_2_-homeobox transcription factor mutants

The seven C_2_H_2_-homeobox transcription factors in the genome of VdLs.17 are VDAG_00465, VDAG_02532, VDAG_02889, VDAG_04660, VDAG_04837, VDAG_04891, and VDAG_07897, named VdChtf1 to VdChtf7, respectively (Fig. S1A and B). To further investigate their functions, we knocked them out using Agrobacterium-mediated genetic transformation (23) and confirmed the knockout by PCR analyses (Fig. S1C through J) using a set of PCR primers (Table S1).

**Fig 1:**
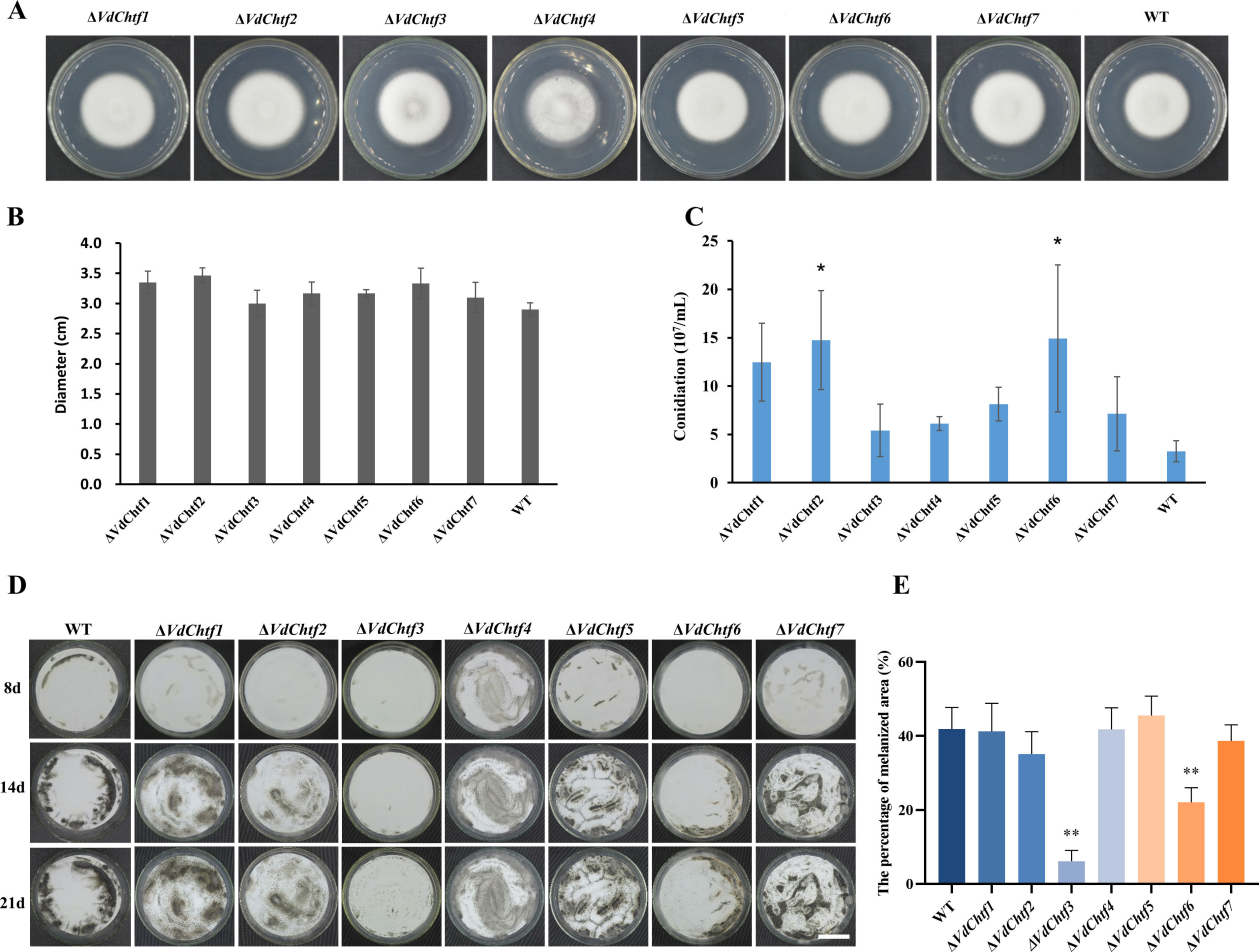
Effects of C_2_H_2_-homeobox transcription factors on the growth and development of *V. dahliae*. (**A**) Growth of C_2_H_2_-homeobox transcription factor mutants and the WT on PDA. (**B**) Statistics of the growth size of colonies in (**A**). (**C**) Statistics of the number of conidia of all the mutants and the WT. Asterisks indicate significant differences (*, *P* < 0.05; **, *P* < 0.01). (**D**) Colony phenotypes of the WT and C_2_H_2_-homeobox transcription factor mutant strains on nitrocellulose membranes overlaid on the basal medium (BM). A conidial suspension (10^6^ conidia/mL) of each strain was dropped onto the membrane and incubated at 25°C. Bar = 3 cm. (**E**) Melanized areas of the WT and the remaining C_2_H_2_-homeobox transcription factor mutant strains were determined using ImageJ after 21 days of incubation. Error bars represent standard deviations based on three independent replicates. Asterisks indicate significant differences (*, *P* < 0.05; **, *P* < 0.01).

**Fig 2:**
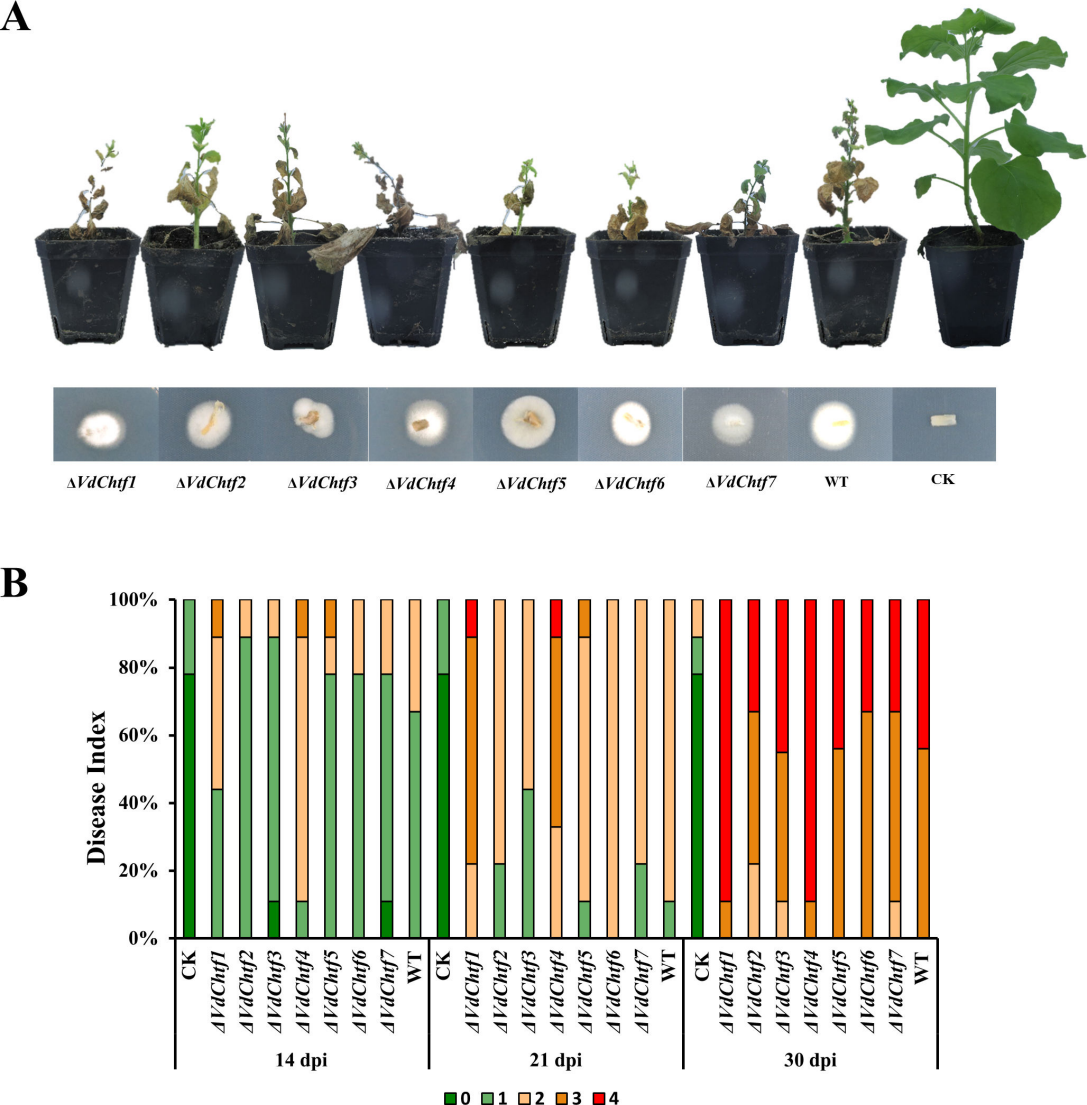
Effects of C_2_H_2_-homeobox transcription factors on the virulence and penetration ability of *V. dahliae*. (**A**) Disease symptoms of *Nicotiana benthamiana*. One-month-old tobacco seedlings were immersed in a conidial suspension (10^6^ conidia/mL) of the WT strain and all of the C_2_H_2_-homeobox transcription factor mutant strains for 10 minutes. The control (CK) was immersed in sterile distilled water and used as the control (CK). After inoculation, all the seedlings were replanted in soil and incubated in a greenhouse for 35 days at 25°C, after which disease symptoms were photographed at 35 days post inoculation (dpi). Below is shown the detection of the pathogen at the stem–root junction of inoculated plants at 35 dpi. (**B**) Bar chart showing the disease index at 35 dpi. Disease indices were visually scored on a scale from 0 (no symptoms) to 4 (more than 85% of the leaves wilted or the whole plant died). The bars represent the percentages of plants at each scale.

**Fig 3:**
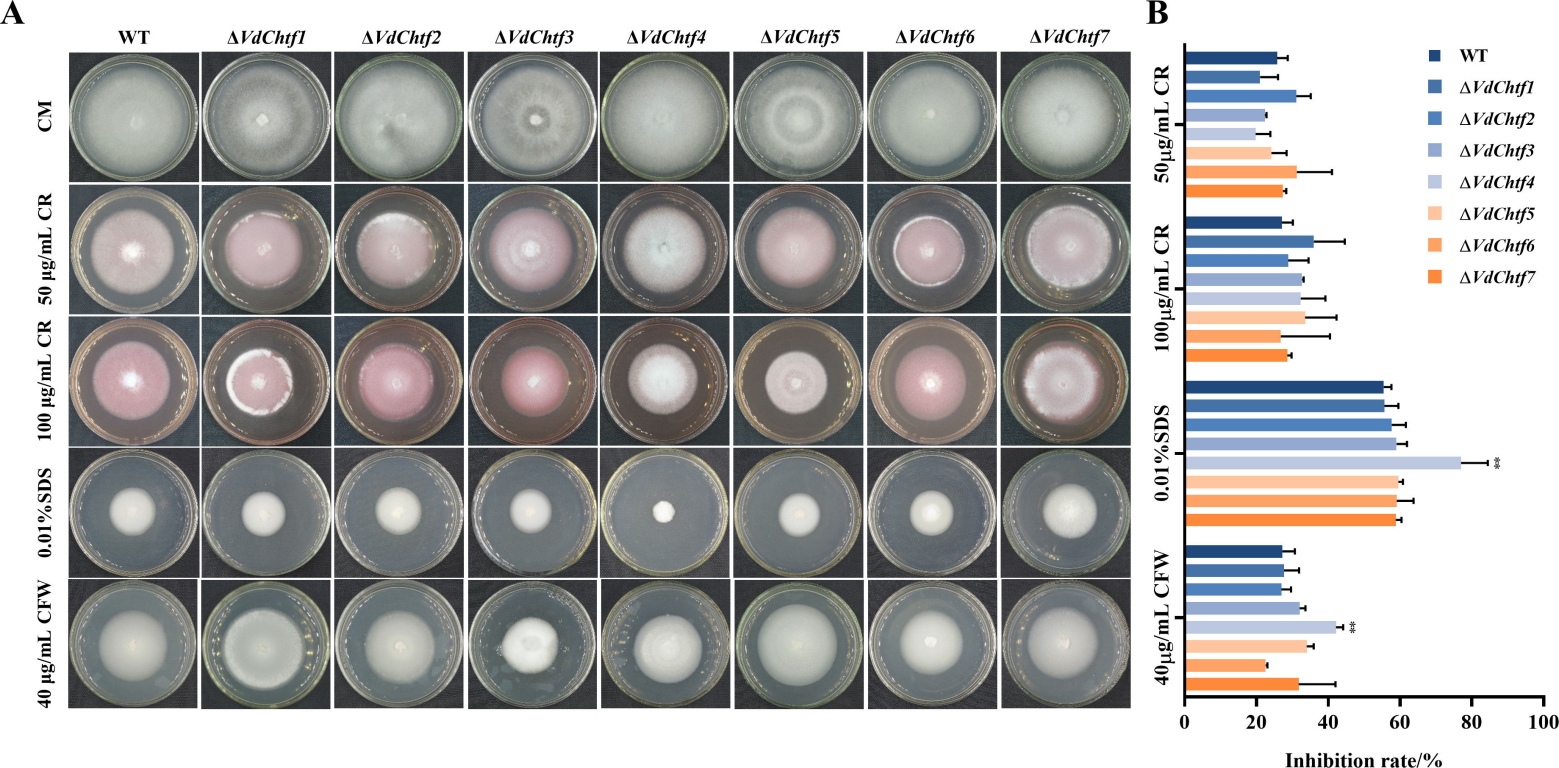
Effects of C_2_H_2_-homeobox transcription factors on the resistance of *V. dahliae* to stress. (**A**) Colonies of WT and C_2_H_2_-homeobox transcription factor mutant strains were grown on complete medium (CM) or CM supplemented with 50 µg/mL Congo red (CR), 100 µg/mL Congo red (CR), and 0.01% SDS or 40 µg/mL calcofluor-white (CFW) for 10 days at 25°C. Bar = 1.5 cm. (**B**) Growth inhibition of the indicated strains by CR, SDS, or CFW. Error bars represent the standard deviation based on three independent replicates (*, *P* < 0.05; **, *P* < 0.01).

The mycelial growth of all the mutants was similar to that of the wild-type (WT) (Fig. 1A and B), while Δ*VdChtf2* and Δ*VdChtf6* generated more conidia on PDA than WT (Fig. 1C). Δ*VdChtf3* and Δ*VdChtf6* showed obvious defects in formation of melanized microsclerotia, with Δ*VdChtf3* showing a more severe phenotype than Δ*VdChtf6* (Fig. 1D and E). All the mutants as well as WT caused tobacco disease and death (Fig. 2A), but according to the disease index statistics, the Δ*VdChtf1* and Δ*VdChtf4* strains exhibited greater virulence (Fig. 2B). To test the penetration ability, all the strains were inoculated on minimal medium (MM) coated with cellophane, and all the mutants, similar to WT, were able to successfully penetrate the cellophane (Fig. S2). By inoculating all strains into CM containing 40 µg/mL CFW, 200 µg/mL CR, and 0.01% SDS, we assessed whether C_2_H_2_-homeobox transcription factors are involved in cell wall regulation. Compared to the WT, Δ*VdChtf4* exhibited increased sensitivity to CFW, CR, and SDS, with inhibition rates higher than the WT by 49.33%, 17%, and 44.67%, respectively. (Fig. 3A and B). The phenotypes of other mutants were indistinguishable as compared with the WT, except for Δ*VdChtf4*. Based on our phenotypic observations of all the *VdChtf* mutants, Δ*VdChtf3* and Δ*VdChtf4* were selected for detailed study because they caused the most severe defects and affected phenotypes associated with important developmental stages in the *V. dahliae* disease cycle.

### VdChtf3 is a major regulator of melanization and microsclerotial formation

**Fig 4:**
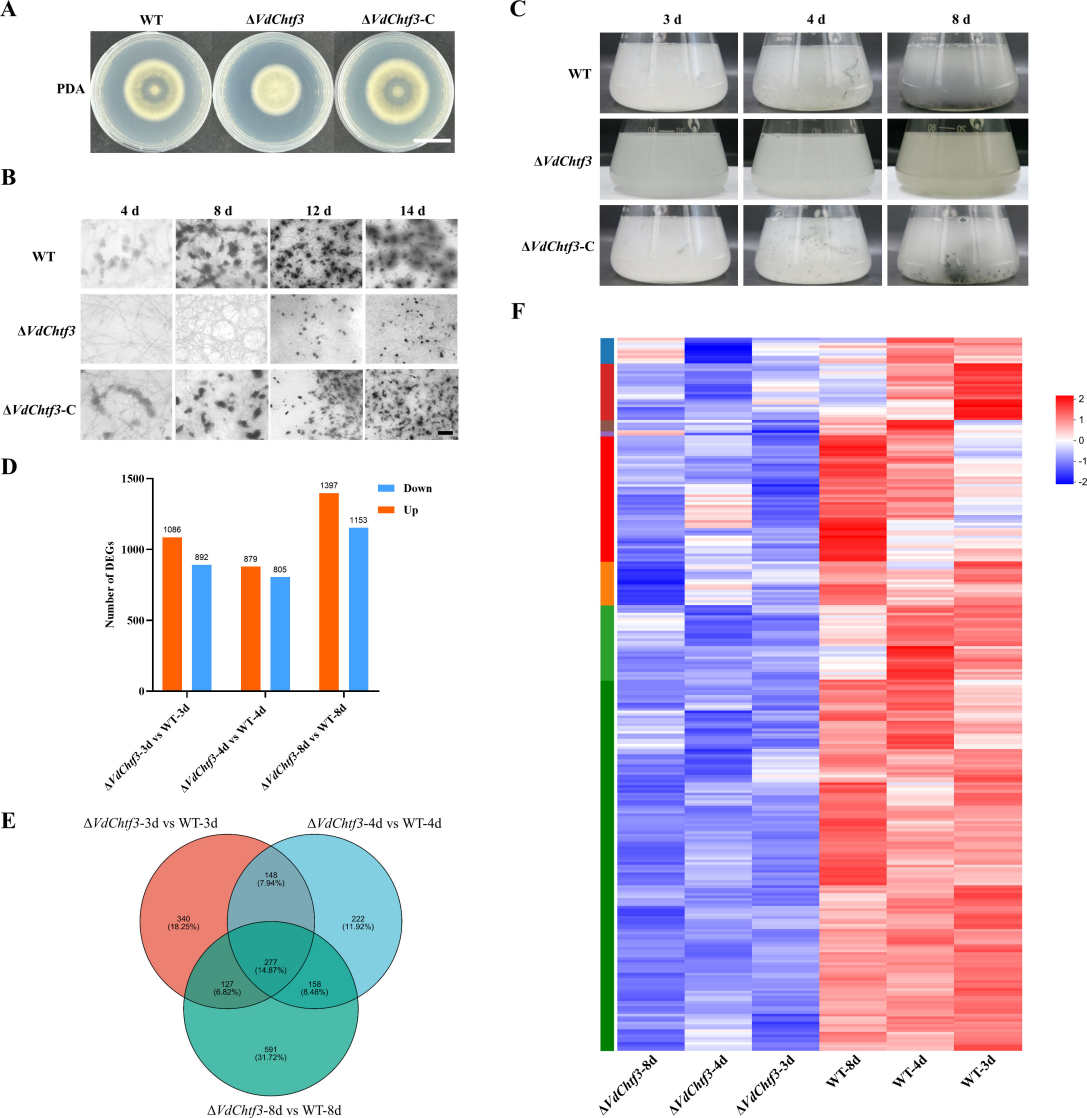
VdChtf3 participated in melanin and microsclerotial formation. (**A**) Conidial morphology of WT, Δ*VdChtf3,* and the complemented strain Δ*VdChtf3*–C on PDA. Bar = 2 cm. (**B**) Microscopic observation of microsclerotial development after 4, 8, 12, and 14 days of incubation. Bar = 100 µm. (**C**) The WT, Δ*VdChtf3,* and Δ*VdChtf3*-C strains were cultured in liquid BM at 25°C and 150 rpm for 3, 4, and 8 days, and samples of WT and Δ*VdChtf3* strains were collected after 3 days for RNA-seq. (**D**) Number of significantly upregulated genes or downregulated genes (*P* < 0.05, false discovery rate <0.05). (**E**) Venn diagram of downregulated DEGs in Δ*VdChtf3* 3 d vs WT 3 d, Δ*VdChtf3* 4 d vs WT 4 d, and Δ*VdChtf3* 8 d vs WT 8 d. (**F**) Hierarchical clustering of expression levels of consistently significantly downregulated genes in the Δ*VdChtf3* mutant during microsclerotial formation. The FPKM value (base-10 logarithm) is the mean of three replicate samples, and the data in this figure are normalized between rows.

The complemented strain Δ*VdChtf3*-C was obtained (Fig. S1K) and showed complete restoration of the defects in microsclerotial formation and melanization observed in Δ*VdChtf3* (Fig. 4A; Fig. S3). To further investigate the role of VdChtf3 in microsclerotial formation, the microsclerotial development of these strains was also observed via microscopy on 4, 8, 12, and 14 days. The Δ*VdChtf3* strain did not form microsclerotia until 12 days, and the melanization area was less than that of WT and complemented strains (Fig. 4B). To further explore whether VdChtf3 affects the morphology of microsclerotia, we observed their microstructure via electron microscopy and found that there was no significant difference between the microsclerotia of the Δ*VdChtf3* mutant and those of WT (Fig. S4). These results suggest that the Δ*VdChtf3* mutant showed a delay in microsclerotial formation and melanin production in *V. dahliae*.

To better understand the role of VdChtf3 in the process of microsclerotial formation, we collected samples from the liquid BM medium after 3, 4, and 8 days (Fig. 4C) and carried out RNA-seq experiments. Transcriptome data showed that 1,978 (1,082 upregulated and 892 downregulated), 1,684 (879 upregulated and 805 downregulated), and 2,550 (1,397 upregulated and 1,153 downregulated) genes were significantly differentially expressed at 3, 4, and 8 days in Δ*VdChtf3* versus WT, respectively (Fig. 4D). The expression of genes associated with microsclerotial formation in Δ*VdChtf3* was delayed compared with WT (Fig. S5). Among all differentially expressed gene (DEGs), 277 genes were consistently significantly downregulated in the Δ*VdChtf3* mutant all the time (Fig. 4E and F; Table S2), which were considered to be activated by VdChtf3 during microsclerotial formation. The GO enrichment analysis showed that these 277 genes were enriched in the extracellular region (GO:0005576), polygalacturonase activity (GO:0004650), hydrolase activity, hydrolyzing O-glycosyl compounds (GO:0004553), flavin adenine dinucleotide binding (GO:0050660), and polysaccharide catabolic process (GO:0000272) (Fig. S6A; Table S3). KEGG enrichment analysis revealed that pentose and glucuronate interconversions (map00040), amino sugar and nucleotide sugar metabolism (map00520), aflatoxin biosynthesis (map00254), tyrosine metabolism (map00350), and valine, leucine, and isoleucine biosynthesis (map00290) were significantly enriched (Fig. S6B; Table S4).

### Pectinases act downstream of VdChtf3 during microsclerotial formation

**Fig 5:**
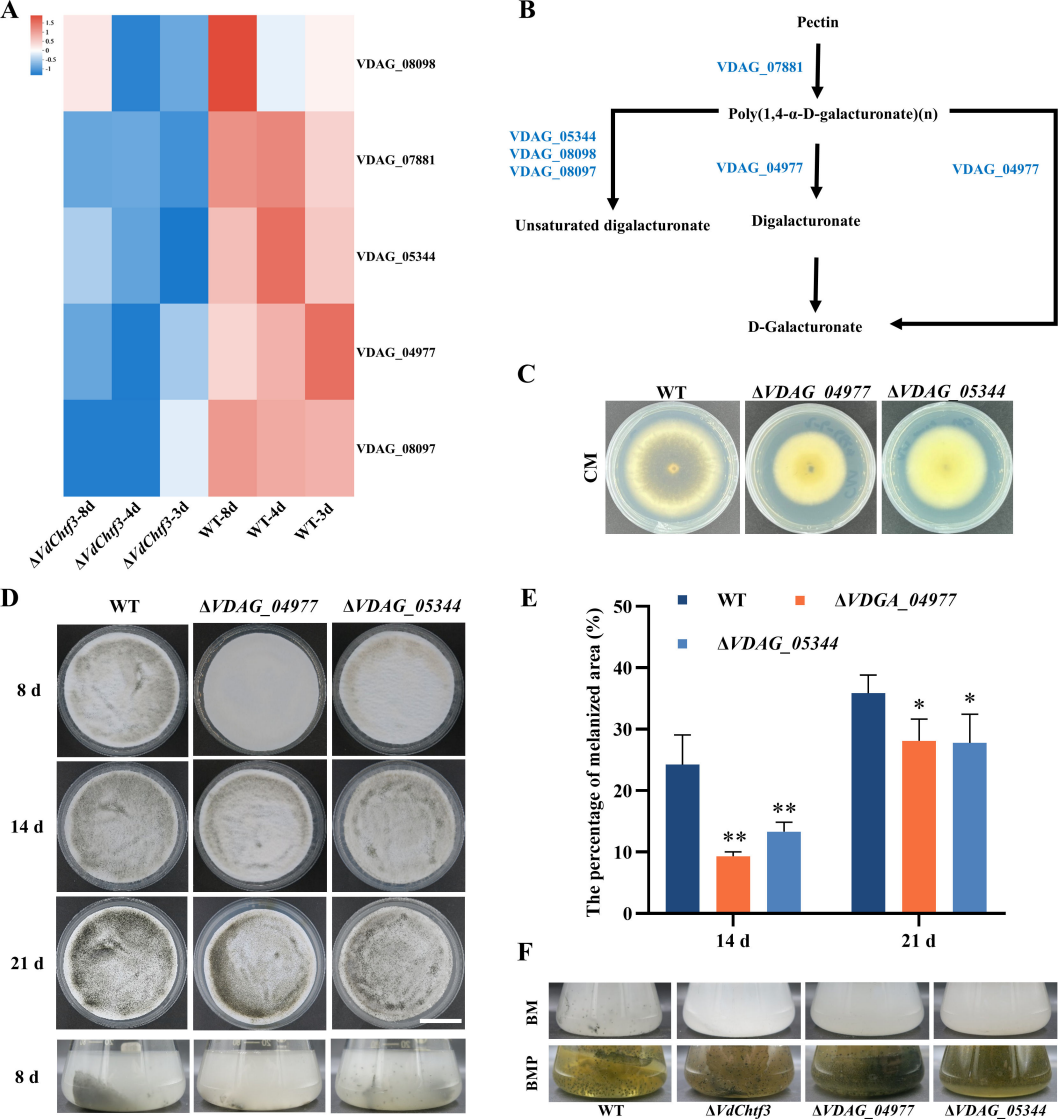
Pectin metabolism genes participate in microsclerotial formation and melanization in *V. dahliae*. (**A**) Heatmap indicating the FPKM values (base-10 logarithm) of the five genes involved in pentose and glucuronate interconversions in the Δ*VdChtf3* and WT strains. The FPKM value is the mean of three replicate samples. (**B**) Schematic diagram of pectin metabolism in *V. dahliae* based on the KEGG network pathway (https://www.genome.jp/kegg/mapper.html). (**C**) Colonies of WT, Δ*VDAG_04977,* and Δ*VDAG_05344* strains grown on complete medium (CM) at 25°C for 10 days. (**D**) Colony phenotypes of WT, Δ*VDAG_04977,* and Δ*VDAG_05344* strains on nitrocellulose membranes overlaid on the basal medium (BM) and in liquid BM. Bar = 3 cm. (**E**) Melanization areas of WT, Δ*VDAG_04977,* and Δ*VDAG_05344* strains were determined using ImageJ after 14 and 21 days of incubation. Error bars represent standard deviations based on three independent replicates. Asterisks indicate significant differences (*, *P* < 0.05; **, *P* < 0.01). (**F**) The colony phenotypes of WT, Δ*VdChtf3*, Δ*VDAG_04977*, and Δ*VDAG_05344* strains were observed after 4 days of growth in liquid BM and BMP.

The pentose and glucuronate interconversion pathway begins with the breakdown of pectin and is responsible for this degradation process by multiple pectinases such as pectinesterase, pectin lyase, and polygalacturonase (24). Among them, five pectinase-encoding DEGs were significantly downregulated in Δ*VdChtf3* (Fig. 5A), which participated in the major steps of pectin metabolism (Fig. 5B). Among them, VDAG_04977 is an endo-polygalacturonase containing a conserved domain of glycosyl hydrolase family 28 (Fig. S7A), and VDAG_05344 is a pectate lyase containing a conserved domain of PelB (Fig. S7B). To test whether these enzymes play important roles in microsclerotial formation, we generated the Δ*VDAG_04977* and Δ*VDAG_05344* strains (Fig. S7C). The two mutants showed a delay in the formation of microsclerotia, although not as significantly defective as Δ*VdChtf3* (Fig. 5C and D). The percentage of the melanized area of Δ*VDAG_04977* and Δ*VDAG_05344* was significantly smaller than that of WT at 14 days, and the difference was reduced at 21 days (Fig. 5E). The results suggested that VdChtf3 activated the expression of *VDAG_04977* and *VDAG_05344* for microsclerotial formation.

VDAG_04977 and VDAG_05344 are both used to metabolize polygalacturonate (PGA) (Fig. 5B), which can induce microsclerotial formation in *V. dahliae* (25). Then, we added PGA to BM, replacing glucose as a carbon source at a comparable concentration and obtained a medium named BMP. The Δ*VdChtf3*, Δ*VDAG_04977*, and Δ*VDAG_05344* strains formed microsclerotia at 4 days when cultured on the BMP medium, suggesting that PGA could restore microsclerotial formation defect owing to gene deletion (Fig. 5F). Besides, the microsclerotia were over-accumulated in the Δ*VDAG_05344* and *VDAG_04977* strains compared with that of WT. These results revealed that the loss of *VDAG_04977* and *VDAG_05344* led to an increase in microscolerotial formation in BMP. These results suggest that regulation of pectinase-encoding genes by VdChtf3 is involved in microsclerotial formation, and due to defects in PGA metabolism, the delayed formation of microsclerotia was restored in the presence of PGA as the carbon source.

### VdChtf4 contributes to full virulence

**Fig 6:**
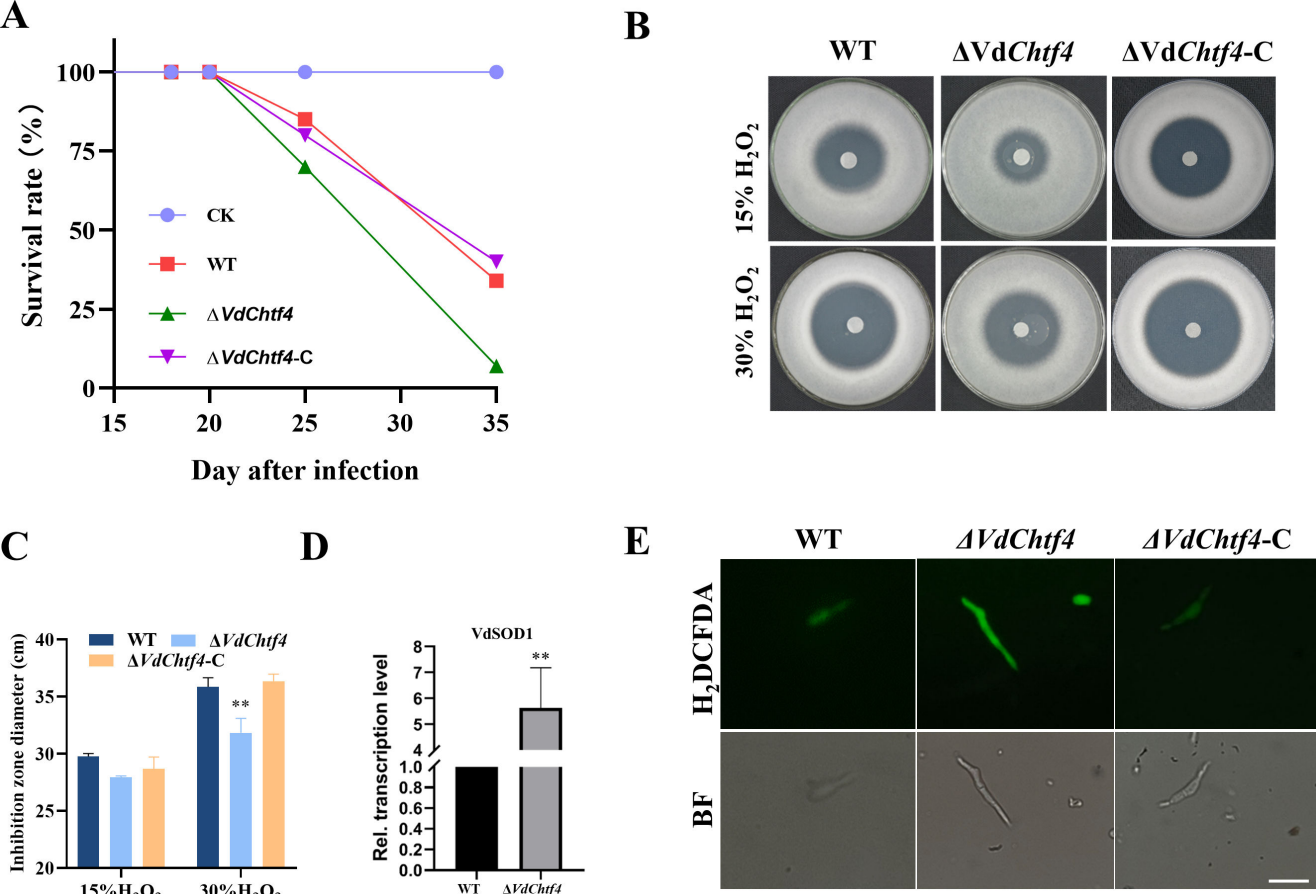
VdChtf4 plays a role in virulence and reactive oxygen species (ROS) resistance in *V. dahliae*. (**A**) Graph showing the survival rate of *Nicotiana benthamiana* inoculated with WT, Δ*VdChtf4,* or Δ*VdChtf4*-C strains. (**B**) Diameter of the inhibition zone under 15% hydrogen peroxide (H_2_O_2_) or 30% hydrogen peroxide (H_2_O_2_) treatment. (**C**) Statistical analysis of the inhibition zone diameter in (**B**). Error bars represent the standard deviation based on three independent replicates (*, *P* < 0.05; **, *P* < 0.01). (**D**) The relative expression levels of *VdSOD1* in the Δ*VdChtf4* strain were examined by RT-qPCR. The average gene expression levels were normalized against that of the *V. dahliae* β-tubulin gene. Error bars represent the standard deviation based on three independent technical replicates (*, *P* < 0.05; **, *P* < 0.01). (**E**) Fluorescence staining after H_2_DCFDA treatment. Hyphae from WT, Δ*VdChtf4,* and Δ*VdChtf4*-C strains were grown in liquid complete medium (CM) for 4 days and treated with 5 µM menadione for 24 hours. H_2_DCFDA was used as a fluorescent dye to visualize the production of hydrogen peroxide (H_2_O_2_). Scale bar = 10 µm.

We obtained the complemented strain of Δ*VdChtf4* (Fig. S1L) and conducted infestation experiments on tobacco (Fig. S8A). The disease index of the Δ*VdChtf4* strain at 35 days post inoculation was significantly greater than that of WT and complemented strains (Fig. S8B). We also found that the Δ*VdChtf4* strain caused tobacco death earlier, compared with WT and complemented strains at 25 dpi (Fig. 6A).

Infection by pathogens is often resisted by host plant immune defenses such as ROS burst. Therefore, we examined the resistance of Δ*VdChtf4* to H_2_O_2_ stress. Although the diameter of the inhibition zone of Δ*VdChtf4* was not significantly different from that of WT and complemented strains when treated with 15% hydrogen peroxide, the diameter of the inhibition zone of Δ*VdChtf4* was significantly smaller than that of WT and complemented strains when treated with 30% hydrogen peroxide (Fig. 6B and C). These results indicated that Δ*VdChtf4* was more resistant to H_2_O_2_ stress. We further verified the expression of genes responding to H_2_O_2_ stress by qPCR and found that the expression of *VdSOD1* was significantly higher after the loss of *VdChtf4* (Fig. 6D). In addition, VdSOD1 affects H_2_O_2_ accumulation when treated with menadione (26), and H_2_DCFDA staining of mycelia after menadione treatment revealed greater H_2_O_2_ accumulation in Δ*VdChtf4* mutant conidia (Fig. 6E), suggesting that Δ*VdChtf4* can handle a greater amount of O^2-^. These results suggest that deletion of *VdChtf4* increased the expression of *VdSOD1*, enhancing the resistance of Δ*VdChtf4* to ROS stress and thus promoting its virulence.

### VdChtf4 is associated with maintaining cell wall integrity

**Fig 7:**
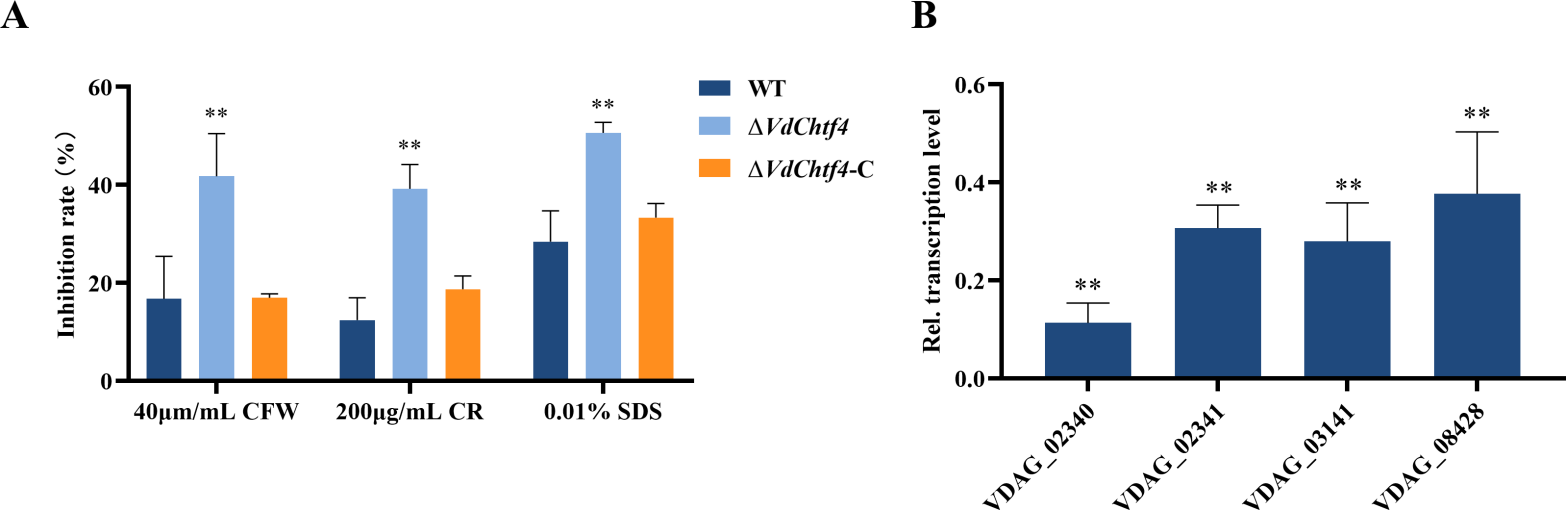
The role of VdChtf4 in cell wall integrity. (**A**) Growth inhibition of the indicated strains by CFW (40 µg/mL), CR (200 µg/mL), or 0.01% SDS. Error bars represent the standard deviation based on three independent replicates (*, *P* < 0.05; **, *P* < 0.01). (**B**) The relative expression levels of genes related to cell wall integrity were examined in the Δ*VdChtf4* strain by RT-qPCR. The average gene expression levels were normalized against the *V. dahliae* β-tubulin gene. Error bars represent the standard deviation based on three independent technical replicates (*, *P* < 0.05; **, *P* < 0.01).

Δ*VdChtf4* also exhibited more significant inhibition than WT strain with high concentrations of Congo Red when compared with WT strain, and Δ*VdChtf4*-C showed complete restoration of the growth defects caused by VdChtf4 under conditions of cell wall stress and cell membrane stress (Fig. 7A; Fig. S8C). This indicated that VdChtf4 was involved in regulating cell wall integrity. We employed qPCR to assess the expression levels of four genes (*VDAG_02340* and *VDAG_02341* encoding 1,3-β-glucan synthase; *VDAG_03141* encoding a chitin synthase; and *VDAG_08428* encoding a glucan synthesis regulatory protein) in both WT and Δ*VdChtf4* strains. The expression of all genes was downregulated in Δ*VdChtf4* (Fig. 7B)*,* suggesting that VdChtf4 may be involved in response to cell wall stress via regulating the expression of genes affecting cell wall integrity.

## DISCUSSION

Transcription factors serve as crucial regulators in fungal development and infection, and their functional analysis often provides new insights into the controlling network that governs fungal development and infection. In this study, we explored the function of seven C_2_H_2_-homeobox transcription factors in *V. dahliae* and found that they play diverse roles in various stress responses, microsclerotial formation, and virulence.

Recent studies have identified several key transcription factors and signaling pathways regulating microsclerotial formation in *V. dahliae* (15, 27–31). Besides microsclerotial formation, these transcription factors affect other biological functions including hyphal growth and virulence in *V. dahliae*. VdChtf3 only participates in microsclerotial formation rather than other biological functions in *V. dahliae*. Therefore, VdChtf3 was considered a possible specific regulator of microsclerotial formation. Transcriptome data revealed that loss of VdChtf3 remarkably downregulated the expression of genes with polygalacturonase activity and genes involved in the pentose and glucoronate interconversion pathway during microsclerotial formation. Among them, two pectinase-encoding genes *VDAG_04977* (endo-polygalacturonase) and *VDAG_05344* (pectate lyase) both took part in the normal formation of microsclerotia. Therefore, VdChtf3 might regulate microsclerotial formation by activating the expressions of *VDAG_04977* and *VDAG_05344* in *V. dahliae*.

VDAG_04977 and VDAG_05344 are cell wall-degrading enzymes (CWDEs), whose roles were mainly thought to focus on the virulence of plant pathogenic fungi in previous studies (32–35). In addition, as observed in the study of *Botrytis cinerea*, the pectinase gene was regulated by Zn(II)2Cys6 transcription factor (36). In *V. dahliae*, certain CWDEs such as α-galactosidase VdGAL4 are related to microsclerotial formation as well as melanin production, suggesting the correlation between CWDEs and microsclerotial formation (37). VDAG_04977 and VDAG_05344 took part in PGA metabolism, and their deletion was likely to cause a higher concentration of PGA, inducing excess microsclerotial formation in *V. dahliae*. The result was consistent with that of the previous study showing that PGA induced the formation of melanized microsclerotia in *V. dahliae* (25). Furthermore, PGA restored microsclerotial formation in the Δ*VdChtf3* strain, revealing that VdChtf3 might affect microsclerotial formation via PGA production, which could be derived from the pectin decomposition or its own metabolic abnormality. However, the Δ*VdChtf3* strain failed to produce microsclerotia excessively, which was different from the Δ*VDAG_04977* and Δ*VDAG_05344* strains. It is probably because the loss of *VdChtf3* upregulated the expression of other genes involved in PGA metabolism simultaneously. Therefore, VdChtf3 might regulate microsclerotial formation by maintaining PGA biosynthesis and metabolism. However, the specific mechanism is still under deep investigation.

ROS burst is the first defense line when plants are attacked by pathogens, which can cross-link plant cell walls to prevent invasion by pathogens (38). Thus, ROS plays a central role in plant immune responses (39). The ability to tolerate or scavenge ROS is often associated with virulence in *V. dahliae*. For example, deletion of *VdDpb4* impaired the ability of *V. dahliae* to repair DNA damage, increasing its sensitivity to ROS and significantly reducing its virulence (40). Deletion of *VdSOD1* and *VdSOD5* led to a decrease in the ROS scavenging ability and reduced the virulence of *V. dahliae* during plant infection (26, 41). In contrast, deletion of the bZip transcription factor *VdMRTF1* significantly enhanced the ROS scavenging ability of *V. dahliae* on tobacco and led to an increase in virulence (42). The deletion of *VdChtf4* significantly upregulated the expression of *VdSOD1* and enhanced the resistance to hydrogen peroxide, indicating that knockout of VdChtf4 enhanced the ability to scavenge superoxide radicals for virulence in *V. dahliae*.

Besides, VdChtf4 was the only member associated with response to cell wall stresses among the seven C_2_H_2_-homeobox transcription factors (Fig. 3A and B; Fig. S5C). The expression of genes encoding chitin synthase and β-glucan synthase, which are involved in cell wall synthesis (43), was decreased in the Δ*VdChtf4* strain, suggesting that VdChtf4 is also involved in regulating cell wall integrity. Although changes in cell wall components may also affect the alteration of mutant virulence, as fungal cell wall components are often thought to trigger the immune defense response of plants (44), whether the regulation of cell wall integrity by VdChtf4 is one of the reasons for the increase in virulence needs to be further studied.

In summary, our research has shown that seven C_2_H_2_-homeobox transcription factors play various roles in *V. dahliae*. VdChtf3 and VdChtf4 cause the most severe defects and affect phenotypes associated with important developmental stages in the *V. dahliae* disease cycle. VdChtf3 modulates the expression of pectinase-encoding genes to influence microsclerotial development. VdChtf4 is associated with cell wall integrity, ROS stress resistance, and increased virulence. These results indicate that the C_2_H_2_-homeobox transcription factors in *V. dahliae* have biological significance for its adaptation to the environment and infection of host plants, contributing to the development of novel strategies for the control of *V. dahliae*.

## MATERIALS AND METHODS

### Fungal strains and culture conditions

*V. dahliae* strain VdLs. 17, isolated from lettuce (11), was used as WT in this study. Conidial suspensions of all strains were stored for long term at −80°C in 30% glycerol. Five-day-old vegetative hyphae collected from liquid compete medium (CM, 50 mL 20 nitrate salts, 1 mL 1000X trace element, 10 g glucose, 2 g peptone, 1 g yeast extract, 1 g casamino acids, and 1 mL vitamin solution per liter) were used for genomic DNA extraction. Selection of strains resistant to the antibiotics hygromycin (Hyg, Sigma, USA) and geneticin (G418 , Sigma, USA) was carried out by plating the respective strains on potato dextrose agar (PDA) plates (200 g potato, 20 g glucose, and 15 g agar per liter) amended with 25 µg/mL hygromycin or 50 µg/mL geneticin, respectively. All strains were grown from a single-spore culture on PDA at room temperature before experimentation, and mycelial masses used for inoculation were obtained using a punch (5 mm).

For analyses of the effects of cell wall inhibitors on mycelial growth, strains were cultivated on CM plates supplemented with 50–200 μg/mL Congo red (CR), 0.01% sodium dodecyl sulfate (SDS), or 40 µg/mL calcofluor-white (CFW) at 25°C for 10 days.

To test oxidative stresses, a 100-µL conidial suspension (10^7^ spores/mL) of each strain was spread onto PDA plates, and filter paper disks containing 5 µL of 15% and 30% hydrogen peroxide (H_2_O_2_) were placed onto the center of each plate. The zone of growth inhibition was measured after 3 dpi.

### Statistics on the number of conidia

Conidia were collected by flooding the PDA plate with 1 mL of sterilized distilled water. The number of conidia was counted by using a hemacytometer under a microscope. Each sample was repeated three times.

### The evaluation of melanin and microsclerotial formation

To observe melanin biosynthesis and microsclerotial formation, 1 mL of a conidial suspension of *V. dahliae* (10^5^ /mL) was coated on a cellulose membrane (Whatman, the UK, Ø = 80 mm; pore size = 0.22 µm), which was overlaid on the solid basal medium (BM, 10 g glucose, 0.2 g NaNO_3_, 0.52 g KCl, 1.52 g KH_2_PO_4_, 0.52 g MgSO_4_·7H_2_O, 3 mM thiamine HCl, 0.1 µM biotin, and 15 g agar per liter) and then cultured at 25°C in dark conditions. The melanin formation was documented by photography after 8, 12, 14, and 21 days; and the melanized area fraction was quantified by ImageJ (45) under default settings (the threshold of all images was 160). Microscopic observations were performed under light microscopy (DM2500 Leica) after incubation for 4, 8, 12, and 14 days. To observe the structure of microsclerotia, the part to be observed was cut from the nitrocellulose membrane, put in the oven, dried at 60°C for 3 hours, and fixed on the scanning electron microscope sample stage, and the microsclerotial structure was observed under the scanning electron microscope. To observe melanin and micorsclerotial formation in liquid BM or in liquid BM with polygalacturonate (Aladdin, Cat: P111886) (BMP, 10 g polygalacturonate, 0.2 g NaNO_3_, 0.52 g KCl, 1.52 g KH_2_PO_4_, 0.52 g MgSO_4_·7H_2_O, 3 mM thiamine HCl, 0.1 µM biotin, 15 g agar per liter, pH adjusted to 7.5), a 1 mL conidial suspension (10^5^ /mL) of each strain was added into liquid BM or BMP and shaken (150 rpm, 25°C) and then documented by photography after 3, 4, and 8 days. All experiments were repeated at least three times.

### Generation of gene deletion mutants or complemented strains

The targeted gene deletion vector pCOM-HYG-VdChtf1 was constructed by inserting two flanking sequences of VdChtf1 on both sides of the HPH gene in the pCOM-HYG vector. An upstream sequence was amplified from *V. dahliae* genomic DNA using primers VdChtf1-5F-F+/R+ (Table S1), which introduced EcoRI cleavage sites into both ends of the fragment. The PCR product was first cloned into the EcoRI site of the pCOM-HYG vector to generate pCOM-HYG-Chtf1-5. A BamHI downstream sequence, amplified using primers VdChtf1-3F-F+/R+ (Table S1), was then inserted into the BamHI site of pCOM-HYG-VdChtf1-5 to generate pCOM-HYG-VdChtf1-53. The same strategy was applied to construct the gene deletion vectors in this study, with the primers (Table S1). To complement the mutants, the DNA fragment carrying the native promoter was amplified with the primers in Table S1. The resulting PCR products were co-transformed with the fragment of G418 by PEG-mediated genetic transformation. The complemented strains were screened with 50 µg/mL of G418. The genomic DNA of the transformants was extracted by using the cetyltrimethylammonium bromide (CTAB) method. The mutants and complemented strains were confirmed by PCR with the primer pairs (Table S1; Fig. S1).

### RNA extraction and gene expression analyses

The total RNA was extracted with the Greenspin total RNA isolation system (ZhuangMeng, China) according to the manufacturer’s instructions. Reverse transcription of RNA using the PrimeScript RT reaction system (ABclonal, China) was performed to obtain cDNA. Reverse transcription–quantitative PCR (RT-qPCR) was performed with the SuperReal PreMix Plus (SYBR Green) using SYBR Green dye and an ABI 7500 real-time PCR system (Applied Biosystems, USA). The *V. dahliae* β-tubulin gene was used as the internal reference for all the qPCR analyses (46), Three biological replicates and three technical replicates were performed for each gene, and the results were analyzed by the 2^-ΔΔCT^ method (47). All the primer sequences are listed in Table S1.

### RNA-seq analysis

For time-series microsclerotial RNA-seq analysis, conidia were harvested from 7-day-old complete medium agar plates, and then 10^7^ conidia/mL of WT or Δ*VdChtf3* was added to the liquid BM and cultured at 25°C, at 150 rpm, in dark conditions. Samples were collected at 3, 4, and 8 days of growth. Total RNA was extracted from the tissue using the Greenspin total RNA isolation system according to the manufacturer’s instructions. Then, RNA quality was determined by 5300 Bioanalyzer (Agilent) and quantified using the ND-2000 (NanoDrop Technologies). Only high-quality RNA samples (OD260/280 = 1.8 to 2.2, OD260/230 > 2.0, RIN > 6.5, 28S:18S > 1.0, >l µg) were used to construct the sequencing library. RNA purification, reverse transcription, library construction, and sequencing were performed at Shanghai Majorbio Bio-pharm Biotechnology Co., Ltd. (Shanghai, China) according to the manufacturer’s instructions (Illumina, San Diego, CA). The raw paired-end reads were trimmed and quality-controlled by fastp (48) with default parameters. Then, clean reads were separately aligned to the reference genome with the orientation mode using HISAT2 software (49). The mapped reads of each sample were assembled by StringTie in a reference-based approach (50). To identify DEGs between two different samples, the expression level of each transcript was calculated according to the transcripts per million reads (TP) method. RSEM was used to quantify gene abundances (51). Essentially, differential expression analysis was performed using the DESeq2 (52). DEGs with |log2FC| ≥ 1 and FDR＜0.05 were considered to be significantly different expressed genes. In addition, functional-enrichment analysis including GO and KEGG was performed to identify which DEGs were significantly enriched in GO terms and metabolic pathways at Bonferroni-corrected *P*-value < 0.05 compared with the whole-transcriptome background. GO functional enrichment and KEGG pathway analysis were carried out by Goatools and Python scipy, respectively.

### The detection of hydrogen peroxide

The WT, Δ*VdChtf4*, and the complemented strains were cultured in the liquid complete medium for 3 days, treated with 5 µM menadione for 24 hours, and stained with 50 µM H_2_DCFDA for 20 minutes. The hyphae and conidia were then washed three times with sterile water and observed under a fluorescence microscope.

### Virulence assays and penetration assays

For virulence assays, 1-month-old tobacco plants (*Nicotiana benthamiana*) and conidial suspensions (10^6^ spores/mL) of the *V. dahliae* strains were used for root-dip inoculation experiments. The roots of tobacco seedlings were immersed in the conidial suspension of each strain for 10 minutes, with sterile water as the control. All the inoculated plants were incubated in a greenhouse at 25°C with a photoperiod of 14-h light/10-h dark for symptom development, and observations were performed every 5 days. The disease was scored at 35 dpi on a scale of 0–4: 0, no wilting; 1, yellowing or wilting of fewer than two leaves; 2, yellowing or wilting of one-fourth leaves; 3, yellowing or wilting of two-third leaves; and 4, more than 85% of the leaves wilted or the whole plant died, and the disease index was determined. Survival rates were scored according to the number of seedlings that had died. Twenty seedlings were inoculated with each strain. At 35 dpi, the sections of the stem–root junction of the tobacco plants were used for re-isolation of the fungus. The steps are consistent with those of our previous research (53).

For the penetration assays on cellophane, conidial suspensions (10^5^ conidia/mL) from each *V. dahliae* strain were introduced at the center of cellophane membranes overlaid on minimal media plates (MM, 6 g NaNO_3_, 1.52 g KH_2_PO_4_, 0.52 g KCl, 0.52 g MgSO_4_.7H_2_O, 20 mM L-glutamic acid, and 15 g agar per liter) and cultivated at 25°C. The cellophane was removed from the plate surface after incubation for 3 and 6 days, and the plate was maintained for another 3 days to observe if there was a colony on MM and thus determine if penetration had occurred before removing the membrane.

### Statistical analysis

The data are presented as the mean ± standard error of the mean. Statistical analyses were performed by ANOVA and Student’s *t*-test (SPSS 16.0). A *P* value < 0.05 (*) and a *P* value < 0.01 (**) were considered to indicate statistical significance.

## Data Availability

The raw sequence and other related data reported in this paper have been deposited in the BIG Data Center, Chinese Academy of Sciences (https://ngdc.cncb.ac.cn/). The accession number of the transcriptomes is (CRA017782).

## SUPPLEMENTAL MATERIAL

Supplemental figures and tables msphere.00409-24-s0001.pdf Fig. S1 to S8; Tables S1 to S4.

ASM does not own the copyrights to Supplemental Material that may be linked to, or accessed through, an article. The authors have granted ASM a non-exclusive, world-wide license to publish the Supplemental Material files. Please contact the corresponding author directly for reuse.
