# Supplementary material for: Transcription factors containing both C2H2 and homeobox domains play different roles in Verticillium dahliae
Source: mSphere. 2024 Aug 27;9(9):e00409-24. doi: 10.1128/msphere.00409-24 (PMC11423567; doi:10.1128/msphere.00409-24)
Supplement: Supplemental figures and tables — Fig. S1 to S8; Tables S1 to S4. [file msphere.00409-24-s0001.pdf]

Figures

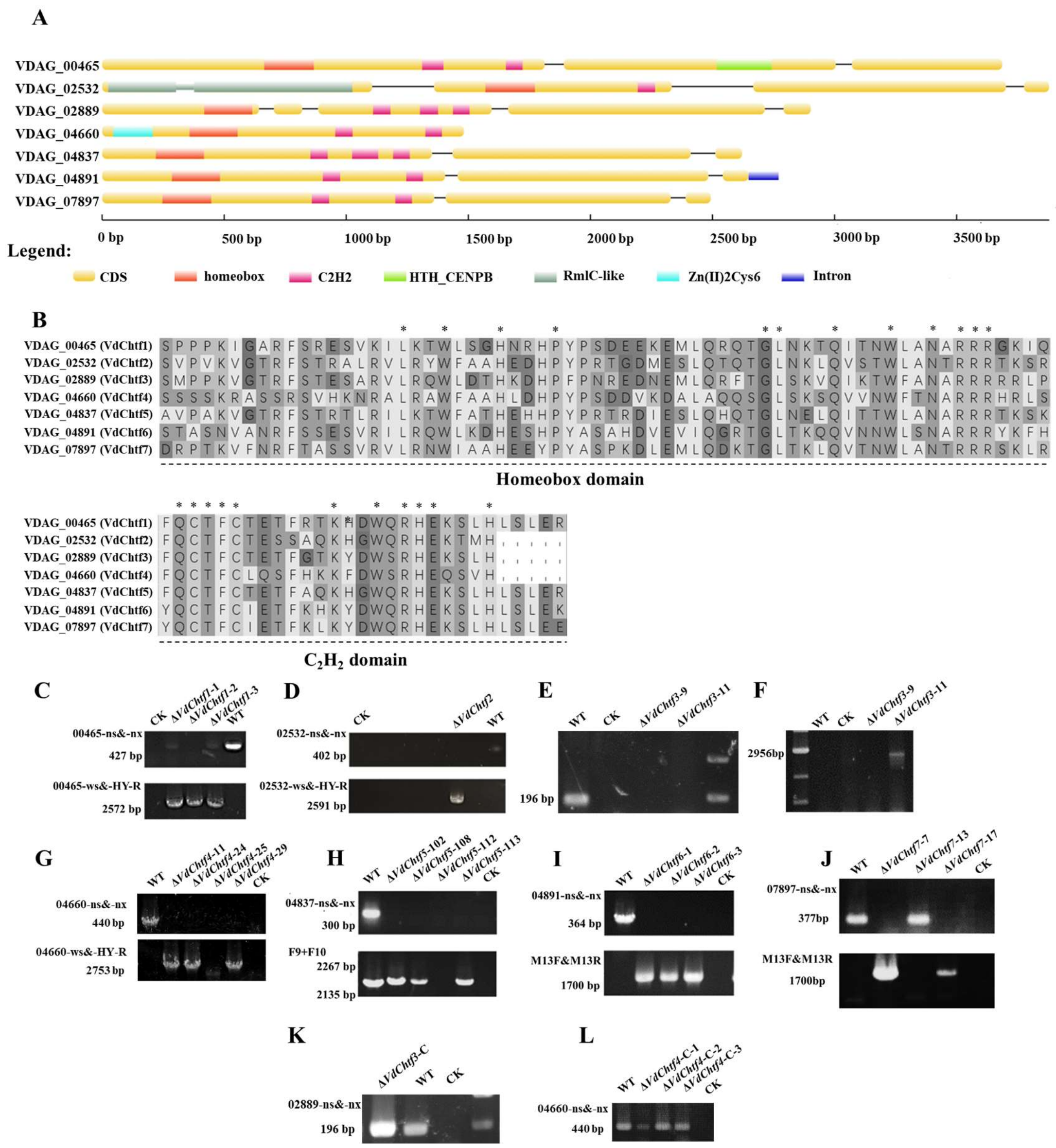

Figure S1. Verification of the C_2H_2-homeobox transcription factor mutants and the complemented strains in *V. dahliae*.

(A) The protein domain of the 7 C2H2-homobox transcription factors. **(B)** Amino acid sequence alignment of the Homeobox domain and C_2H_2 domain of seven C_2H_2-homeobox transcription factor. **(C-J)** PCR assays of the WT and mutant strains were performed with primers. The sizes of the PCR products are shown in the figures. **(K)** PCR validation of the VdChtf3 complemented strains. **(L)** PCR validation of the

VdChtf4 complemented strains.

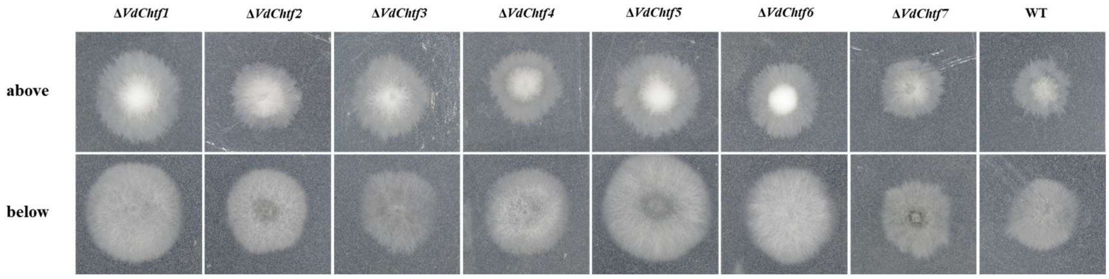

Figure S2. Colonies of WT and mutant strains grown on minimal medium (MM) overlaid with (top panel) or without (bottom panel) a cellophane membrane. Strains were grown on MM plates overlaid with a cellophane membrane at 25 °C for 6 days and incubated for another 3 days after the membrane was removed.

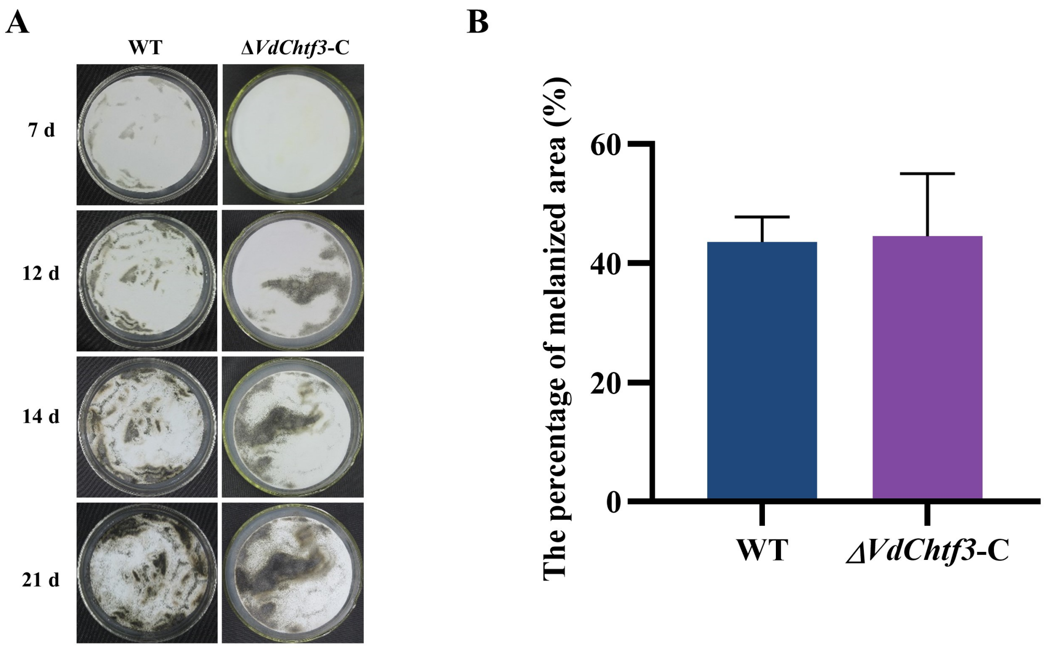

Figure S3. Phenotypes of Δ*VdChtf3*-C in terms of microsclerotial formation and melanization.

(A) Colony phenotypes of the WT and Δ*VdChtf3*-C strains on nitrocellulose membranes overlaid on basal medium (BM). **(B)** The melanization areas of the WT strain and the complemented strain Δ*VdChtf3*–C were determined using ImageJ after 21 days of incubation. Error bars represent standard deviations based on three independent replicates.

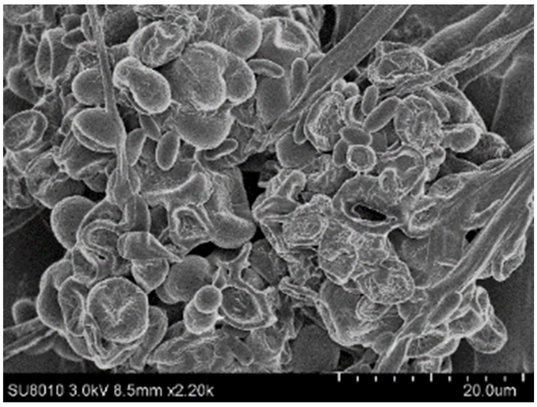

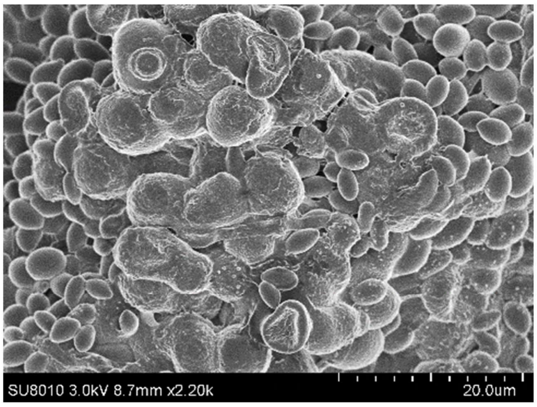

WT **Δ*VdChtf3***

Figure S4. Morphology of 14-day-old microsclerotia incubated on BM observed by scanning electron microscopy.

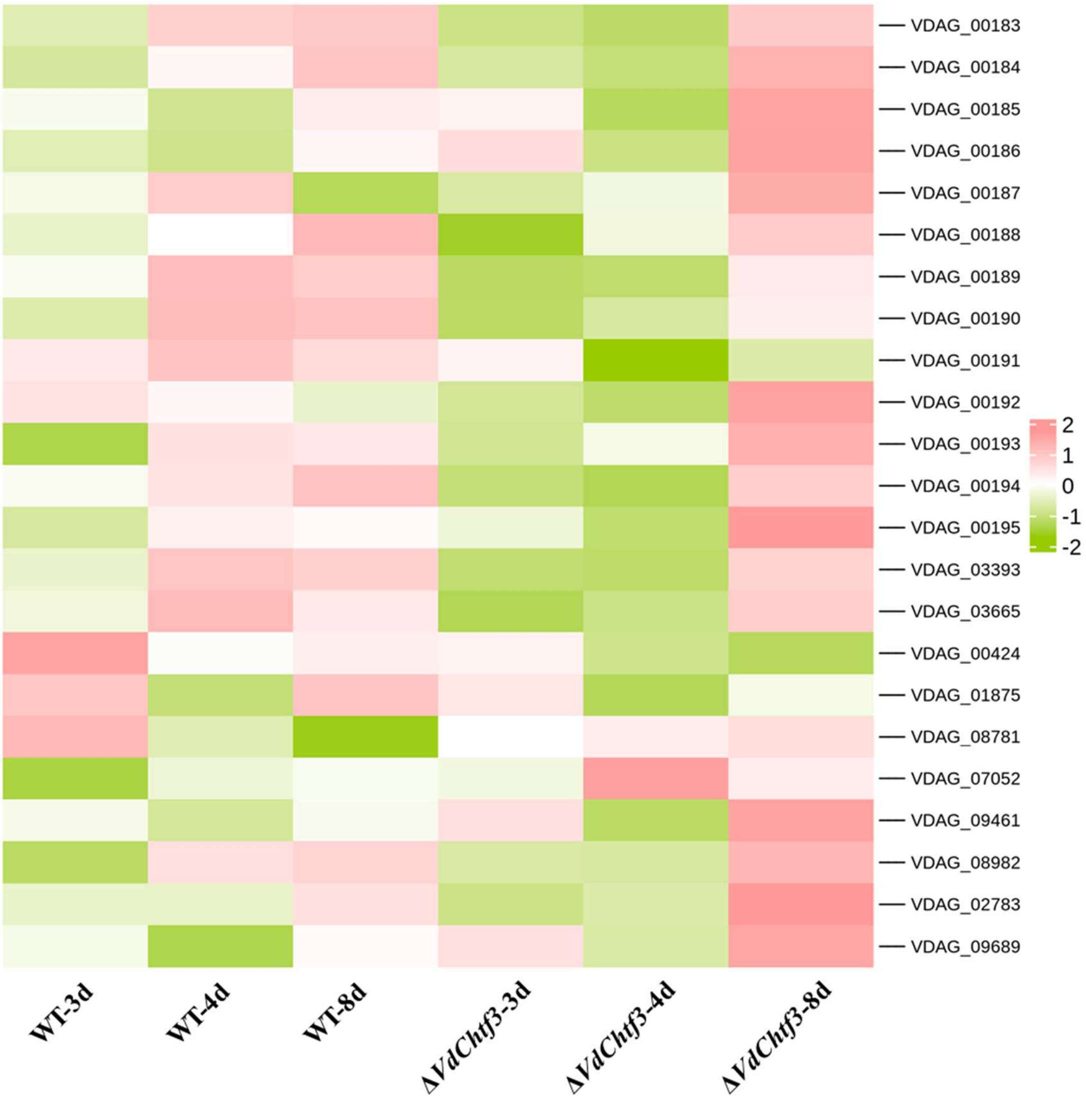

Figure S5. The expression of key genes that have been reported during microsclerotial formation

Heatmap indicating the FPKM value (base-10 logariyhm) of genes that have been reported during microsclerotial formation in in *V. dahliae*. The average FPKM value was from three biological repetitions.

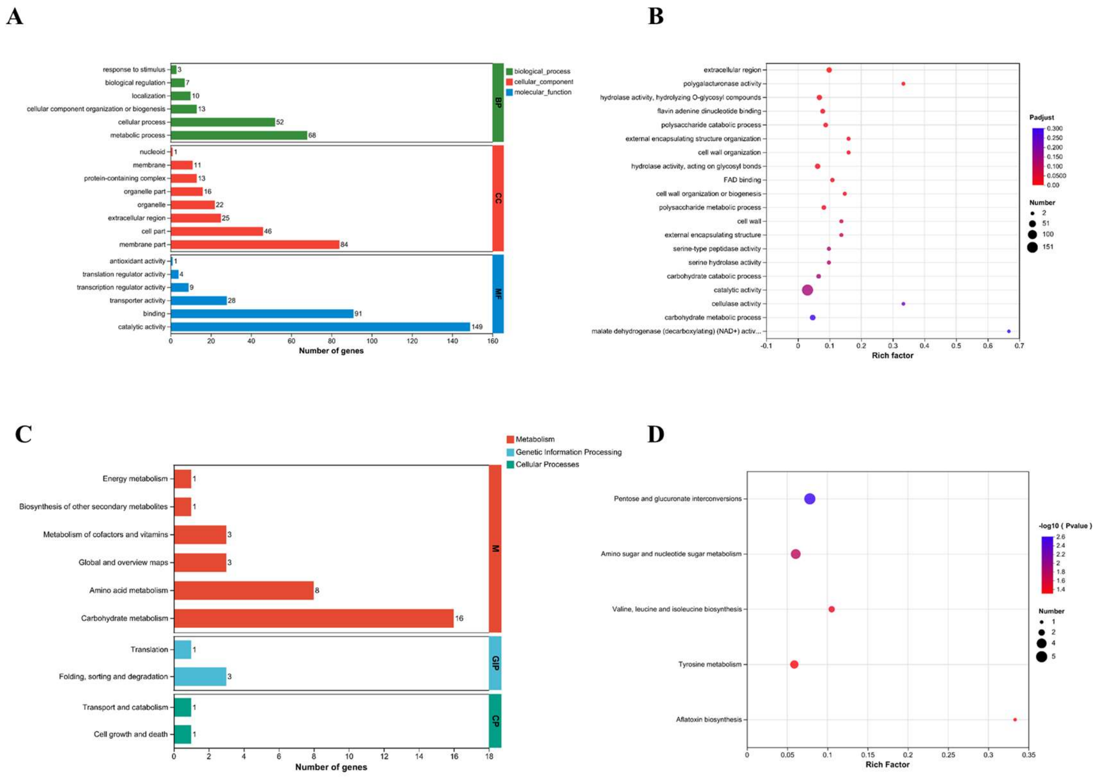

Figure S6. Annotation analysis of differentially expressed genes.

(A) Gene Ontology annotation analysis of consistently significantly downregulated genes in Δ*VdChtf3* mutant during microsclerotial formation. **(B)** Gene Ontology enrichment of consistently significantly downregulated genes in Δ*VdChtf3* mutant during microsclerotial formation. **(D)** Kyoto Encyclopedia of Genes and Genomes pathway classification of consistently significantly downregulated genes in Δ*VdChtf3* mutant during microsclerotial formation. **(E)** Kyoto Encyclopedia of Genes and Genomes pathway enrichment of consistently significantly downregulated genes in Δ*VdChtf3* mutant during microsclerotial formation.

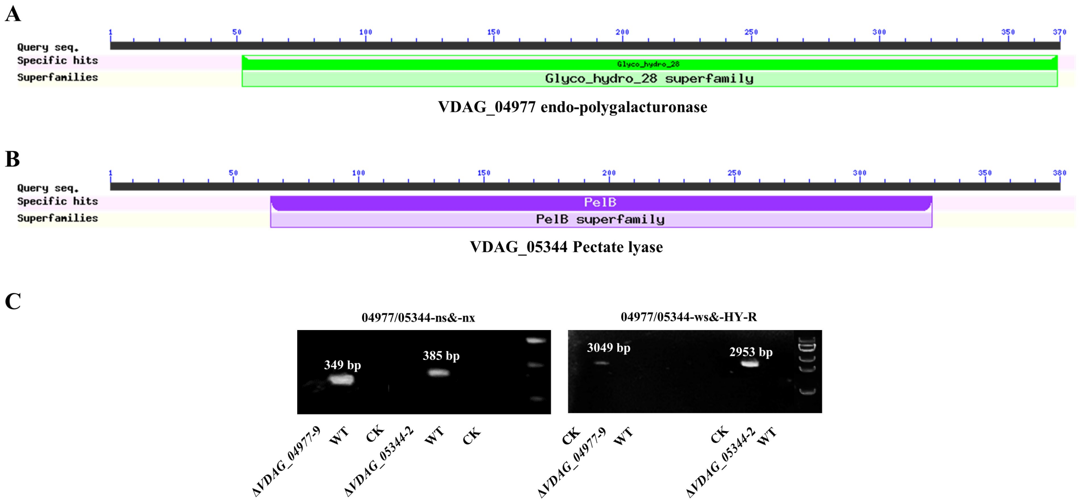

Figure S7. Verification of the Δ*VDAG_04977* and Δ*VDAG_05344* mutants in *V. dahliae*.

(A) Conserved domain analysis of VDAG_04977 via NCBI Conserved Domain Search.

(B) Conserved domain analysis of VDAG_05344 via NCBI Conserved Domain Search.

(C) PCR assays of the WT and mutants were performed with primers. The sizes of the PCR products are shown in the figures.

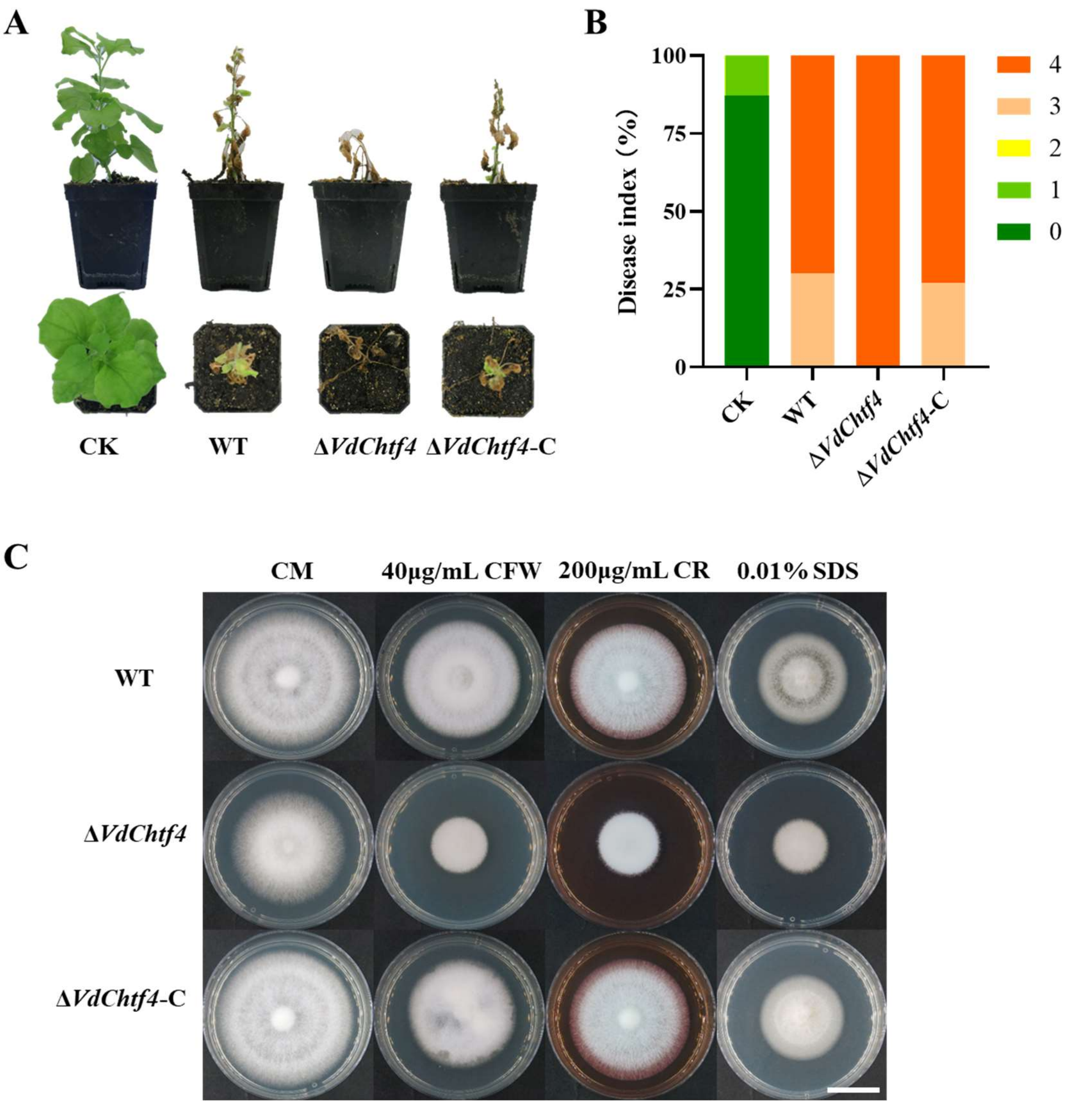

Figure S8. The virulence and growth phenotypes of the Δ*VdChtf4*-C strain under stress.

(A) Disease symptoms of *Nicotiana benthamiana* inoculated with WT, Δ*VdChtf4* and Δ*VdChtf4*-C strains. **(B)** Bar chart showing the disease index at 35 days post inoculation (dpi). Disease indices were visually scored on a scale from 0 (no symptoms) to 4 (completely wilted or dead). The bars represent the percentages of plants with each score. **(C)** Colonies of the WT, Δ*VdChtf4* and Δ*VdChtf4*-C strains were grown on complete medium (CM) or CM supplemented with 40 μg/mL calcofluor white (CFW), 200 μg/mL Congo red (CR), or 0.01% SDS for 10 days at 25 °C. Bar = 2 cm.

Tables

Table S1. List of primers used in this study.

Primer names	Primer sequence	Primer function
VdChtf1-5F-F	AGGCGGATTCGCCATATC	5' flanking primers
VdChtf1-5F-R	CTGAACAGGCGGCACAGT	
VdChtf1-3F-F	GATTCTCTGGTGCCCAATGC	3' flanking primers
VdChtf1-3F-R	GAAGAGGCCGCGCCTTAT	
VdChtf1-5F-F+	CGTCACCAGCCCCTGGGTTGAATTCAGGCGGATTCGCCATATC	5' flanking primers with cap of pCOM-Hyg
VdChtf1-5F-R+	AAATGCTCCTTCAATATCAGAATTCCTGAACAGGCGGCACAGT	
VdChtf1-3F-F+	ACCAGAGCTCGGTACCCGGGGATCCGATTCTCTGGTGCCCAATGC	3' flanking primers with cap of pCOM-hyg
VdChtf1-3F-R+	CCTGCAGGTCGACTCTAGAGGATCCGAAGAGGCCGCGCCTTAT	
VdChtf1-ns	GCTCAGGGACCTCTTCATGT	Internal primer/ RT primer
VdChtf1-nx	GCGGCTGTAGATGTGGATTG	
VdChtf1-ws	CCTCCGAATGAACCGCAAAT	External primers
VdChtf1-wx	CTGCTTCGTGTCCTTGCTTT	
VdChtf2-5F-F	GATCTCGCACATTCCAGAC	5' flanking primers
VdChtf2-5F-R	TGGGATGGGATGGGTAAGTCCT	
VdChtf2-3F-F	TGCCTATGATCTCTCAAGAC	3' flanking primers
VdChtf2-3F-R	GAGCTGCTGCGGCGTGACTGA	
VdChtf2-5F-F+	CGTCACCAGCCCCTGGGTTGAATTCGATCTCGCACATTCCAGAC	5' flanking primers with cap of pCOM-Hyg
VdChtf2-5F-R+	AAATGCTCCTTCAATATCAGAATTCTGGGATGGGATGGGTAAGTCCT	
VdChtf2-3F-F+	ACCAGAGCTCGGTACCCGGGGATCCTGCCTATGATCTCTCAAGAC	3' flanking primers with cap of pCOM-hyg

VdChtf2-3F-R+	CCTGCAGGTCGACTCTAGAGGATCCGAGCTGCTGCGGCGTGACTGA	
VdChtf2-ns	TATCCACCTCGGAGAACTT	Internal primer/ RT primer
VdChtf2-nx	TGCCATCACCGTTAGTTAC	
VdChtf2-ws	GAGGGCGCATAATGTGGAAG	External primers
VdChtf2-wx	TCCTCCAGCATCAATCGTGT	
VdChtf3-5F-F	TCGACACTTACAAGGGATGATG	5' flanking primers
VdChtf3-5F-R	GACTCGAAGTTCCTTGGTGGTT	
VdChtf3-3F-F	TGATTACGTGGAGCGGAAACAG	3' flanking primers
VdChtf3-3F-R	TACAGAAGTGAGTCCGTTCTAA	
VdChtf3-5F-F+	CGTCACCAGCCCCTGGGTTGAATTCTCGACACTTACAAGGGATGATG	
VdChtf3-5F-R+	AAATGCTCCTTCAATATCAGAATTCGACTCGAAGTTCCTTGGTGGTT	5' flanking primers with cap of pCOM-hyg
VdChtf3-3F-F+	ACCAGAGCTCGGTACCCGGGGATCCTGATTACGTGGAGCGGAAACAG	3' flanking primers with cap of pCOM-hyg
VdChtf3-3F-R+	CCTGCAGGTCGACTCTAGAGGATCCTACAGAAGTGAGTCCGTTCTAA	
VdChtf3-ns	AACGACGCAAACTATCACCG	Internal primer/ RT primer
VdChtf3-nx	CCCATCATGCTCAACTGA	
VdChtf3-ws	AGCCTCGCTGTTACCTTGAC	External primers
VdChtf3-wx	GTGAGTTAGGCCTGAGGTGT	
C3C-F	TCCGTGTCGATGGGTAAACA	Native promoter and ORF of VdChtf3
C3C-R	TCGGCGCTATATCCTGGAAG	
VdChtf4-5F-F	CTGCTGTCCGAGGCTATGC	5' flanking primers
VdChtf4-5F-R	CGTGTCGATGTCCAGTCGTG	
VdChtf4-3F-F	GTATATGGCGATGTTGCC	3' flanking primers
VdChtf4-3F-R	CAACGTGGCAGACACGTCT	
VdChtf4-5F-F+	CGTCACCAGCCCCTGGGTTGAATTCCTGCTGTCCGAGGCTATGC	
VdChtf4-5F-R+	AAATGCTCCTTCAATATCAGAATTCCGTGTCGATGTCCAGTCGTG	5' flanking primers with cap of pCOM-hyg

VdChtf4-3F-F+	ACCAGAGCTCGGTACCCGGGGATCCGTATATGGCGATGTTGCC	3' flanking primers with cap of pCOM-hyg
VdChtf4-3F-R+	CCTGCAGGTCGACTCTAGAGGATCCCAACGTGGCAGACACGTCT	
VdChtf4-ns	GTATCCGTCCGATGATGTAA	Internal primer/ RT primer
VdChtf4-nx	CGTAGTGGTTGCTTGTAGA	
VdChtf4-ws	GTGACCAAGTTCAAGGCCAG	External primers
VdChtf4-wx	TGAACTTCTGCTCTCGTCGT	
C4C-F		
C4C-R		
VdChtf5-5F-F	GTCATGGCTAGAAACTC	5' flanking primers
VdChtf5-5F-R	GATACATTTTGCTACAGCAG	
VdChtf5-3F-F	TCATATCAGACGAGGACATC	3' flanking primers
VdChtf5-3F-R	TTACATTCACAGTGGTATGC	
VdChtf5-5F-F+	CGTCACCAGCCCCTGGGTTGAATTCGTCATGGCTAGAAACTC	
VdChtf5-5F-R+	AAATGCTCCTTCAATATCAGAATTCGATACATTTTGCTACAGCAG	5' flanking primers with cap of pCOM-hyg
VdChtf5-3F-F+	ACCAGAGCTCGGTACCCGGGGATCCTTACATTCACAGTGGTATGC	3' flanking primers with cap of pCOM-hyg
VdChtf5-3F-R+	CCTGCAGGTCGACTCTAGAGGATCCTTACATTCACAGTGGTATGC	
VdChtf5-ns	CAAGGACCATCTCAGACAA	Internal primer/ RT primer
VdChtf5-nx	GAAGGAGTAAGGAGTAGCATTA	
F9	GCACGCTAAGTTACTACAC	External primers for VdChtf5
F10	GGTCTTCCTCCAGATTACAT	
VdChtf6-5F-F	TGACGAGATGTAGGTGCGTA	5' flanking primers
VdChtf6-5F-R	GACGGACCGTCTGTGCTA	
VdChtf6-3F-F	GCTTGTCACGATGCATAG	3' flanking primers
VdChtf6-3F-R	CCGAGGTGAACCAGTTCT	
VdChtf6-5F-F+	CGTCACCAGCCCCTGGGTTGAATTCTGACGAGATGTAGGTGCGTA	

VdChtf6-5F-R+	AAATGCTCCTTCAATATCAGAATTCGACGGACCGTCTGTGCTA	5' flanking primers with cap of pCOM-hyg
VdChtf6-3F-F+	ACCAGAGCTCGGTACCCGGGGATCCGCTTGTCACGATGCATAG	3' flanking primers with cap of pCOM-hyg
VdChtf6-3F-R+	CCTGCAGGTCGACTCTAGAGGATCCCCGAGGTGAACCAGTTCT	
VdChtf6-ns	TTCATTACGAGCGCATAT	Internal primer/ RT primer
VdChtf6-nx	TCAAGGTCACAATCATCG	
VdChtf7-5F-F	TACTTGGAGTTCCAGGAATG	5' flanking primers
VdChtf7-5F-R	TCTCGTGCTTCAGATAAACTG	
VdChtf7-3F-F	GCCGACAGCAAGCATCGTAC	3' flanking primers
VdChtf7-3F-R	TATCGTCCTTACTCCAGAGCAT	
VdChtf7-5F-R+	CGTCACCAGCCCCTGGGTTGAATTCTACTTGGAGTTCCAGGAATG	
VdChtf7-5F-R+	AAATGCTCCTTCAATATCAGAATTCTCTCGTGCTTCAGATAAACTG	5' flanking primers with cap of pCOM-hyg
VdChtf7-3F-F+	ACCAGAGCTCGGTACCCGGGGATCCGCCGACAGCAAGCATCGTAC	3' flanking primers with cap of pCOM-hyg
VdChtf7-3F-R+	CCTGCAGGTCGACTCTAGAGGATCCTATCGTCCTTACTCCAGAGCAT	
VdChtf7-ns	AAGGACCTCGAAATGCTCCA	Internal primer/ RT primer
VdChtf7-nx	CATGCACGGATCAGACTTGG	
VdChtf7-ws	TCGTGATGACTTAGCGGACG	External primers
VdChtf7-wx	GCGTCATTTTCGCCGATTGA	
04977-5F-F	TGGGCGTCAGATTCTTGAGT	5' flanking primers
04977-5F-R	GCAGTTCACTCATCGTCGAC	
04977-3F-F	ACCACGACAAGCTAGCTCTT	3' flanking primers
04977-3F-R	CAAATCAACGGTCGCTGCTA	
04977-5F-F+	CGTCACCAGCCCCTGGGTTGAATTCTGGGCGTCAGATTCTTGAGT	
04977-5F-R+	AAATGCTCCTTCAATATCAGAATTCGCAGTTCACTCATCGTCGAC	5' flanking primers with cap of pCOM-hyg
04977-3F-F+	ACCAGAGCTCGGTACCCGGGGATCCACCACGACAAGCTAGCTCTT	3' flanking primers with cap of pCOM-hyg
04977-3F-R+	CCTGCAGGTCGACTCTAGAGGATCCCAAATCAACGGTCGCTGCTA	

04977-ns	CTCAACAACATTGCTGTCC	Internal primer
04977-nx	CGTCGATGAGCTGAAGGTTG	
05344-5F-F	CGTAGTGGCTGAGATGGCTA	5' flanking primers
05344-5F-R	AAGAGGGAGAGGGCAGAAAC	
05344-3F-F	TTGAAGAGACCAGGGCGAAT	3' flanking primers
05344-3F-R	AGGTCGCCGTAGATGTACTG	
05344-5F-F+	CGTCACCAGCCCCTGGGTTGAATTCCGTAGTGGCTGAGATGGCTA	
05344-5F-R+	AAATGCTCCTTCAATATCAGAATTCAAGAGGGAGAGGGCAGAAAC	5' flanking primers with cap of pCOM-hyg
05344-3F-F+	ACCAGAGCTCGGTACCCGGGGATCCTTGAAGAGACCAGGGCGAAT	3' flanking primers with cap of pCOM-hyg
05344-3F-R+	CCTGCAGGTCGACTCTAGAGGATCCAGGTCGCCGTAGATGTACTG	
05344-ns	ACGCCTGTGACATTGGTTTC	Internal primer
05344-nx	TCGTAGAAGTCCTTGCCGTG	
02340-rt-F	CTCGAGCCACCGATATGGAC	RT primer for VDAG_02340
02340-rt-R	GGAAACCAAACTTGGCGGTC	
02341-rt-F	TCATCCGTGGAGGTCGTA	RT primer for VDAG_02341
02341-rt-R	CCTGTCAAGAGGGAGTTGTG	
03141-rt-F	TCATGGCGAAGAAATGGC	RT primer for VDAG_03141
03141-rt-R	CGTAGAAACCGCAGTAGACC	
08428-rt-F	CGAGGAGTTGGTGCAAGAGT	RT primer for VDAG_08428
08428-rt-R	GTTATTGCCACCCCAATCGC	
VdSOD1-rt-F	CGTTATTGGCCGTACCGTCG	RT primer for VdSOD1
VdSOD1-rt-R	AGTTAGAGATGCCGATGACACC	
VdBt-up	AGCTCACCCAGCAGATGTTC	RT primer for β-tubulin
VdBt-down	TCGACCTCCTTCATGGCAAC	
M13F	CGCCAGGGTTTTCCCAGTCACGAC	M13 primes

M13R	AGCGGATAACAATTTCACACAGGA	
EN-F	AATATCACGGGTAGCCAACG	G418 split marker primers
GE-R	TGAATGAACTGCAGGACGAG	
YG-F	CGTTGCAAGACCTGCCTGAA	hygromycin split marker primers
HY-R	GGATGCCTCCGCTCGAAGTA	

Table S2. Consistently significantly downregulated genes in ΔVdChtf3 mutant during microsclerotial formation.

Gene_name	Gene_description		Gene_name	Gene_description	
VDAG_04805	-		VDAG_09205	Glycoside hydrolase, catalytic core	
VDAG_04831	Chromo domain-like		VDAG_09223	-	
VDAG_05491	Heavy metal transport/detoxification protein		VDAG_08866	Esterase, SGNH hydrolase-type	
VDAG_04913	-		VDAG_00055	-	
VDAG_04916	-		VDAG_09275	-	
VDAG_04977	Pectin lyase fold/virulence factor		VDAG_09288	Glycoside hydrolase, catalytic core	
VDAG_05505	-		VDAG_09311	-	
VDAG_04994	Cytochrome P450		VDAG_09322	-	
VDAG_05506	MFS general substrate transporter		VDAG_09327	UBA-like	
VDAG_05017	-		VDAG_09340	-	
VDAG_05042	Concanavalin A-like lectin/glucanase		VDAG_09345	FAD-binding, type 2	
VDAG_05048	MFS general substrate transporter		VDAG_09366	Pectin lyase fold/virulence factor	
VDAG_05117	-		VDAG_09376	-	
VDAG_05160	Protein kinase-like		VDAG_09382	Glucose-methanol-choline oxidoreductase, N-terminal	
VDAG_05178	Ankyrin		VDAG_09492	2OG-Fe(II) oxygenase	
VDAG_05262	-		VDAG_09493	RNA polymerase II-associated protein 1, N-terminal	
VDAG_05534	-		VDAG_09506	Survival protein SurE-like phosphatase/nucleotidase	
VDAG_05613	Peptidase, cysteine peptidase active site		VDAG_09526	FAD-binding, type 2	
VDAG_05635	-		VDAG_09527	-	
VDAG_05636	Fungal transcriptional regulatory protein, N-terminal		VDAG_09528	Thioredoxin-like fold	
VDAG_05637	MFS general substrate transporter		VDAG_09529	Protein kinase ATP binding, conserved site	

VDAG_05652	Peptidase S41		VDAG_09533	NAD(P)-binding	
VDAG_05830	Cytochrome P450		VDAG_09534	Malonyl-CoA ACP transacylase, ACP-binding	
VDAG_05835	NAD(P)-binding		VDAG_09541	Cytochrome P450	
VDAG_05839	NAD(P)-binding		VDAG_09560	Glycoside hydrolase, catalytic core	
VDAG_05864	Phosphatidylethanolamine-binding protein PEBP		VDAG_09574	HI0933-like protein	
VDAG_05866	-		VDAG_09764	-	
VDAG_05916	MFS general substrate transporter		VDAG_09794	-	
VDAG_05973	-		VDAG_09802	Acyl carrier protein-like	
VDAG_05983	-		VDAG_09805	MFS general substrate transporter	
VDAG_06010	NAD(P)-binding		VDAG_09613	Glycoside hydrolase, catalytic core	
VDAG_06016	Pyruvate/Phosphoenolpyruvate kinase, catalytic core		VDAG_09615	WW/Rsp5/WWP	
VDAG_06026	-		VDAG_09616	-	
VDAG_06031	-		VDAG_00617	-	
VDAG_06032	Cytochrome P450		VDAG_00618	Glucose-methanol-choline oxidoreductase, N-terminal	
VDAG_06042	Cytochrome cd1-nitrite reductase-like, C-terminal haem d1		VDAG_09898	Zinc finger, AN1-type	
VDAG_00189	Cupredoxin	*VdLAC,* Zhang, 2017	VDAG_00635	Glycosyl transferase, family 8	
VDAG_00019	Barwin-related endoglucanase		VDAG_01687	Esterase, SGNH hydrolase-type	
VDAG_00190	Malonyl-CoA ACP transacylase, ACP-binding	*VdPKS,* Zhang, 2017	VDAG_01718	Zinc finger, C2H2-type	
VDAG_06084	-		VDAG_01806	-	
VDAG_06120	-		VDAG_01843	-	
VDAG_06130	-		VDAG_01862	Signal recognition particle, SRP54 subunit, helical bundle	
VDAG_06133	Fumarate reductase/succinate dehydrogenase flavoprotein, N-terminal		VDAG_01905	-	

VDAG_06199	Barwin-related endoglucanase		VDAG_02008	-	
VDAG_06204	Haem peroxidase		VDAG_02043	Fungal chitosanase	
VDAG_06212	Alpha/beta hydrolase fold-1		VDAG_02045	Ctr copper transporter	
VDAG_06233	-		VDAG_02067	-	
VDAG_06234	NUDIX		VDAG_02103	-	
VDAG_06240	Phytanoyl-CoA dioxygenase		VDAG_02106	Methyltransferase type 12	
VDAG_06254	Cellulose-binding region, fungal		VDAG_02110	Amino acid permease, conserved site	
VDAG_06277	NAD(P)-binding		VDAG_02125	Concanavalin A-like lectin/glucanase	
VDAG_06283	MFS general substrate transporter		VDAG_02181	Cytochrome P450	
VDAG_06822	Methyltransferase type 12		VDAG_02204	-	
VDAG_06885	Ankyrin		VDAG_02222	MFS general substrate transporter	
VDAG_06931	-		VDAG_02229	Pyridoxal phosphate-dependent transferase, major region	
VDAG_07123	Endoribonuclease L-PSP/chorismate mutase-like		VDAG_02278	Ankyrin	
VDAG_07142	Glycoside hydrolase/deacetylase, beta/alpha-barrel		VDAG_00070	Peptidase S8 and S53, subtilisin, kexin, sedolisin	
VDAG_07171	-		VDAG_00071	MFS general substrate transporter	
VDAG_07185	Glycoside hydrolase, catalytic core		VDAG_10084	-	
VDAG_07235	MFS general substrate transporter		VDAG_10092	AAA ATPase, conserved site	
VDAG_07238	Pectin lyase fold/virulence factor		VDAG_10093	-	
VDAG_07265	-		VDAG_10367	-	
VDAG_07266	Glucose-methanol-choline oxidoreductase, N-terminal		VDAG_02345	-	
VDAG_07282	-		VDAG_02492	Rhamnose mutarotase	
VDAG_07306	Peptidase S10, serine carboxypeptidase		VDAG_00785	-	
VDAG_07359	Zinc finger, C2H2-type		VDAG_02708	-	
VDAG_07367	Proteinase inhibitor, propeptide		VDAG_02712	-	

VDAG_07399	Pyruvate/Phosphoenolpyruvate kinase, catalytic core		VDAG_02727	Pyridoxal phosphate-dependent transferase, major region	
VDAG_07406	Cellulose-binding region, fungal		VDAG_02735	-	
VDAG_07421	Carbonic anhydrase, eukaryotic		VDAG_02827	FAD-binding, type 2	
VDAG_07423	Acyl-CoA N-acyltransferase		VDAG_02828	-	
VDAG_00307	MFS general substrate transporter		VDAG_02889	Homeodomain-like	
VDAG_07610	-		VDAG_02906	-	
VDAG_07680	Thioesterase superfamily		VDAG_02909	-	
VDAG_07681	ABC transporter-like		VDAG_02917	-	
VDAG_07701	-		VDAG_05292	Fungal transcriptional regulatory protein, N-terminal	
VDAG_07727	Glycoside hydrolase/deacetylase, beta/alpha-barrel		VDAG_02959	Glycoside hydrolase, family 61	
VDAG_07766	-		VDAG_02980	-	
VDAG_07847	Ethyl tert-butyl ether degradation EthD		VDAG_10474	Proteinase inhibitor, propeptide	
VDAG_07881	Pectin lyase fold/virulence factor		VDAG_10515	-	
VDAG_07920	MFS general substrate transporter		VDAG_10527	Chitin-binding, type 1	
VDAG_07922	Calycin-like		VDAG_03142	WD40 repeat-like	
VDAG_07923	NAD(P)-binding		VDAG_03143	Glycoside hydrolase, catalytic core	
VDAG_07924	-		VDAG_03287	-	
VDAG_07925	Fungal transcriptional regulatory protein, N-terminal		VDAG_03309	Arsenical-resistance protein ACR3	
VDAG_07926	-		VDAG_03325	Cupin, RmlC-type	
VDAG_07928	Malonyl-CoA ACP transacylase, ACP-binding		VDAG_03340	-	
VDAG_07969	Concanavalin A-like lectin/glucanase		VDAG_03347	ATP-grasp fold	
VDAG_08012	-		VDAG_03369	-	
VDAG_08061	MFS general substrate transporter		VDAG_03374	-	
VDAG_08097	Pectin lyase fold/virulence factor		VDAG_05344	Pectin lyase fold/virulence factor	

VDAG_08098	Pectin lyase fold/virulence factor		VDAG_03405	Beta-Ig-H3/fasciclin	
VDAG_08100	Peptidase S8 and S53, subtilisin, kexin, sedolisin		VDAG_05354	Peptidase M, neutral zinc metallopeptidases, zinc-binding site	
VDAG_08104	-		VDAG_03485	NAD(P)-binding	
VDAG_08106	Flavin-containing monooxygenase FMO		VDAG_03495	FAD-binding, type 2	
VDAG_08125	AAA+ ATPase, core		VDAG_03496	-	
VDAG_08141	Glucose-methanol-choline oxidoreductase, N-terminal		VDAG_03523	CoA-transferase family III	
VDAG_08151	-		VDAG_03534	-	
VDAG_08152	-		VDAG_03544	-	
VDAG_08213	-		VDAG_03616	NAD(P)-binding	
VDAG_08217	-		VDAG_03708	Glycoside hydrolase, family 43, endo-1, 5-alpha-L-arabinosidase	
VDAG_08218	-		VDAG_03713	-	
VDAG_08223	Acyl-CoA N-acyltransferase		VDAG_03755	-	
VDAG_08230	FAD-binding, type 2		VDAG_03811	MFS general substrate transporter	
VDAG_08256	-		VDAG_03844	-	
VDAG_08282	SMP-30/Gluconolaconase/LRE-like region		VDAG_03887	FAD-binding, type 2	
VDAG_00845	-		VDAG_03892	Quinonprotein alcohol dehydrogenase-like	
VDAG_00915	-		VDAG_03893	-	
VDAG_01109	-		VDAG_03894	Alpha/beta hydrolase fold-3	
VDAG_01212	Glycosyl transferase, family 8		VDAG_03905	Peptidase S8 and S53, subtilisin, kexin, sedolisin	
VDAG_01226	Coproporphyrinogen III oxidase		VDAG_04030	-	
VDAG_01252	-		VDAG_04031	-	
VDAG_01323	NAD(P)-binding		VDAG_04032	Metallophosphoesterase	

VDAG_00436	Pleckstrin-like		VDAG_05411	Cobalamin (vitamin B12) biosynthesis CobW-like, C-terminal	
VDAG_00450	Hly-III related proteins		VDAG_04084	HSP20-like chaperone	
VDAG_01637	Aminotransferase, class IV		VDAG_04106	Fumarylacetoacetase, C-terminal-related	
VDAG_08371	DNA/RNA helicase, ATP-dependent, DEAH-box type, conserved site		VDAG_04125	-	
VDAG_08514	Homeodomain-like		VDAG_04148	-	
VDAG_08589	NAD(P)-binding		VDAG_04150	Protein kinase-like	
VDAG_08619	-		VDAG_04196	-	
VDAG_08644	Zinc finger, C2H2-type		VDAG_04227	Zinc finger, C2H2-type	
VDAG_08681	-		VDAG_04291	Peptidase aspartic, catalytic	
VDAG_08723	Rhodanese-like		VDAG_04306	-	
VDAG_08728	WD40 repeat-like		VDAG_04335	-	
VDAG_09063	Glycoside hydrolase-type carbohydrate-binding		VDAG_04336	Concanavalin A-like lectin/glucanase	
VDAG_09071	MFS general substrate transporter		VDAG_04407	Cell surface antigen	
VDAG_09072	Endoribonuclease L-PSP/chorismate mutase-like		VDAG_05448	Peptidase M, neutral zinc metallopeptidases, zinc-binding site	
VDAG_09081	-		VDAG_04434	-	
VDAG_09082	Coenzyme A transferase active site		VDAG_04444	Pyridine nucleotide-disulphide oxidoreductase, NAD-binding region	
VDAG_09123	Trimeric LpxA-like		VDAG_05460	NADH:flavin oxidoreductase/NADH oxidase, N-terminal	
VDAG_09125	-		VDAG_05461	-	
VDAG_09138	Ferric reductase-like transmembrane component, N-terminal		VDAG_04551	-	

VDAG_09144	Fungal transcriptional regulatory protein, N-terminal		VDAG_04634	-	
VDAG_09148	Basic-leucine zipper (bZIP) transcription factor		VDAG_04686	Pyridine nucleotide-disulphide oxidoreductase, NAD-binding region	
VDAG_09149	Zinc finger, RING-type		VDAG_04719	-	
VDAG_09186	-		VDAG_04737	-	
VDAG_09190	-		VDAG_04738	FAD-binding, type 2	
VDAG_09200	Glycoside hydrolase, catalytic core		VDAG_04754	Chondroitin AC/alginate lyase	
VDAG_09204	-				

Table S3. Gene Ontology (GO) annotation and enrichment analysis of differentially expressed genes listed in Table S2.

Gene Ontology annotation			
GO ID	Description	Term Type	num
GO:0065007	biological regulation	biological_process	7
GO:0008152	metabolic process	biological_process	68
GO:0071840	cellular component organization or biogenesis	biological_process	13
GO:0009987	cellular process	biological_process	52
GO:0051179	localization	biological_process	10
GO:0050896	response to stimulus	biological_process	3
GO:0032991	protein-containing complex	cellular_component	13
GO:0044425	membrane part	cellular_component	84
GO:0044422	organelle part	cellular_component	16
GO:0043226	organelle	cellular_component	22
GO:0016020	membrane	cellular_component	11
GO:0005576	extracellular region	cellular_component	25
GO:0009295	nucleoid	cellular_component	1
GO:0044464	cell part	cellular_component	46
GO:0045182	translation regulator activity	molecular_function	4
GO:0140110	transcription regulator activity	molecular_function	9
GO:0016209	antioxidant activity	molecular_function	1
GO:0005215	transporter activity	molecular_function	28
GO:0005488	binding	molecular_function	91
GO:0003824	catalytic activity	molecular_function	149

Gene Ontology enrichment						
GO ID	Term Type	Description	Ratio_in_study	Ratio_in_pop	Rich factor	Pvalue
GO:0005576	CC	extracellular region	25/ 216	253/ 8471	0.098814	4.36E-07
GO:0004650	MF	polygalacturonase activity	6/ 216	18/ 8471	0.333333	3.68E-06
GO:0004553	MF	hydrolase activity, hydrolyzing O-glycosyl compounds	23/ 216	339/ 8471	0.067847	1.65E-05
GO:0050660	MF	flavin adenine dinucleotide binding	18/ 216	230/ 8471	0.078261	2.20E-05
GO:0000272	BP	polysaccharide catabolic process	15/ 216	171/ 8471	0.087719	2.91E-05
GO:0045229	BP	external encapsulating structure organization	8/ 216	50/ 8471	0.16	3.35E-05
GO:0071555	BP	cell wall organization	8/ 216	50/ 8471	0.16	3.35E-05
GO:0016798	MF	hydrolase activity, acting on glycosyl bonds	24/ 216	386/ 8471	0.062176	4.47E-05
GO:0071949	MF	FAD binding	11/ 216	101/ 8471	0.108911	4.88E-05
GO:0071554	BP	cell wall organization or biogenesis	8/ 216	54/ 8471	0.148148	5.95E-05
GO:0005976	BP	polysaccharide metabolic process	15/ 216	183/ 8471	0.081967	6.40E-05
GO:0005618	CC	cell wall	7/ 216	51/ 8471	0.137255	0.000284
GO:0030312	CC	external encapsulating structure	7/ 216	51/ 8471	0.137255	0.000284
GO:0008236	MF	serine-type peptidase activity	9/ 216	92/ 8471	0.097826	0.000535
GO:0017171	MF	serine hydrolase activity	9/ 216	92/ 8471	0.097826	0.000535
GO:0016052	BP	carbohydrate catabolic process	15/ 216	229/ 8471	0.065502	0.000741
GO:0003824	MF	catalytic activity	151/ 216	4975/ 8471	0.030352	0.000744
GO:0008810	MF	cellulase activity	3/ 216	9/ 8471	0.333333	0.001226
GO:0005975	BP	carbohydrate metabolic process	28/ 216	600/ 8471	0.046667	0.001736
GO:0004471	MF	malate dehydrogenase (decarboxylating) (NAD+) activity	2/ 216	3/ 8471	0.666667	0.001909
GO:0004470	MF	malic enzyme activity	2/ 216	3/ 8471	0.666667	0.001909
GO:0043115	MF	precorrin-2 dehydrogenase activity	2/ 216	4/ 8471	0.5	0.003754
GO:0016491	MF	oxidoreductase activity	49/ 216	1301/ 8471	0.037663	0.003954

GO:0004497	MF	monooxygenase activity	10/ 216	153/ 8471	0.065359	0.005677
GO:0019354	BP	siroheme biosynthetic process	2/ 216	5/ 8471	0.4	0.006152
GO:0046156	BP	siroheme metabolic process	2/ 216	5/ 8471	0.4	0.006152
GO:0004315	MF	3-oxoacyl-[acyl-carrier-protein] synthase activity	3/ 216	16/ 8471	0.1875	0.007163
GO:0016615	MF	malate dehydrogenase activity	2/ 216	6/ 8471	0.333333	0.009073
GO:0004252	MF	serine-type endopeptidase activity	5/ 216	53/ 8471	0.09434	0.010937
GO:0031221	BP	arabinan metabolic process	2/ 216	7/ 8471	0.285714	0.01249
GO:0031222	BP	arabinan catabolic process	2/ 216	7/ 8471	0.285714	0.01249
GO:0006779	BP	porphyrin-containing compound biosynthetic process	3/ 216	24/ 8471	0.125	0.022301
GO:0033014	BP	tetrapyrrole biosynthetic process	3/ 216	25/ 8471	0.12	0.024876
GO:0009821	BP	alkaloid biosynthetic process	1/ 216	1/ 8471	1	0.025499
GO:0035835	BP	indole alkaloid biosynthetic process	1/ 216	1/ 8471	1	0.025499
GO:0035834	BP	indole alkaloid metabolic process	1/ 216	1/ 8471	1	0.025499
GO:0033212	BP	iron import into cell	1/ 216	1/ 8471	1	0.025499
GO:0033215	BP	reductive iron assimilation	1/ 216	1/ 8471	1	0.025499
GO:1901072	BP	glucosamine-containing compound catabolic process	3/ 216	26/ 8471	0.115385	0.027605
GO:0006032	BP	chitin catabolic process	3/ 216	26/ 8471	0.115385	0.027605
GO:0046348	BP	amino sugar catabolic process	3/ 216	26/ 8471	0.115385	0.027605
GO:0006778	BP	porphyrin-containing compound metabolic process	3/ 216	26/ 8471	0.115385	0.027605
GO:0006026	BP	aminoglycan catabolic process	3/ 216	27/ 8471	0.111111	0.030487
GO:0033013	BP	tetrapyrrole metabolic process	3/ 216	27/ 8471	0.111111	0.030487
GO:0009057	BP	macromolecule catabolic process	15/ 216	333/ 8471	0.045045	0.030927
GO:0006783	BP	heme biosynthetic process	2/ 216	12/ 8471	0.166667	0.036102
GO:0006487	BP	protein N-linked glycosylation	2/ 216	14/ 8471	0.142857	0.04815
GO:0042168	BP	heme metabolic process	2/ 216	14/ 8471	0.142857	0.04815

GO:0005785	CC	signal recognition particle receptor complex	1/ 216	1/ 8471	1	0.025499
GO:0098799	CC	outer mitochondrial membrane protein complex	2/ 216	13/ 8471	0.153846	0.041962
GO:0004312	MF	fatty acid synthase activity	3/ 216	20/ 8471	0.15	0.013531
GO:0016747	MF	acyltransferase activity, transferring groups other than amino-acyl groups	9/ 216	151/ 8471	0.059603	0.015003
GO:0031177	MF	phosphopantetheine binding	3/ 216	22/ 8471	0.136364	0.01761
GO:0004175	MF	endopeptidase activity	9/ 216	157/ 8471	0.057325	0.018891
GO:0072341	MF	modified amino acid binding	3/ 216	24/ 8471	0.125	0.022301
GO:0016787	MF	hydrolase activity	65/ 216	1989/ 8471	0.03268	0.022744
GO:0016977	MF	chitosanase activity	1/ 216	1/ 8471	1	0.025499
GO:0050048	MF	L-leucine:2-oxoglutarate aminotransferase activity	1/ 216	1/ 8471	1	0.025499
GO:0052654	MF	L-leucine transaminase activity	1/ 216	1/ 8471	1	0.025499
GO:0052655	MF	L-valine transaminase activity	1/ 216	1/ 8471	1	0.025499
GO:0052656	MF	L-isoleucine transaminase activity	1/ 216	1/ 8471	1	0.025499
GO:0008716	MF	D-alanine-D-alanine ligase activity	1/ 216	1/ 8471	1	0.025499
GO:0004568	MF	chitinase activity	3/ 216	26/ 8471	0.115385	0.027605
GO:0016854	MF	racemase and epimerase activity	2/ 216	12/ 8471	0.166667	0.036102
GO:0008233	MF	peptidase activity	18/ 216	434/ 8471	0.041475	0.040229
GO:0008061	MF	chitin binding	3/ 216	31/ 8471	0.096774	0.043521

Table S4. Kyoto Encyclopedia of Genes and Genomes (KEGG) annotation and enrichment analysis of differentially expressed genes in Table S2.

KEGG pathway annotation				
First Category	Second Category	pathway ID	Description	numbers
Metabolism	Global and overview maps	map01240	Biosynthesis of cofactors	3
Metabolism	Amino acid metabolism	map00270	Cysteine and methionine metabolism	2
Metabolism	Carbohydrate metabolism	map00040	Pentose and glucuronate interconversions	5
Metabolism	Amino acid metabolism	map00330	Arginine and proline metabolism	1
Metabolism	Amino acid metabolism	map00250	Alanine, aspartate and glutamate metabolism	1
Metabolism	Amino acid metabolism	map00350	Tyrosine metabolism	3
Metabolism	Biosynthesis of other secondary metabolites	map00254	Aflatoxin biosynthesis	1
Metabolism	Carbohydrate metabolism	map00630	Glyoxylate and dicarboxylate metabolism	1
Metabolism	Metabolism of cofactors and vitamins	map00770	Pantothenate and CoA biosynthesis	2
Metabolism	Carbohydrate metabolism	map00650	Butanoate metabolism	1
Metabolism	Metabolism of cofactors and vitamins	map00860	Porphyrin and chlorophyll metabolism	1
Metabolism	Amino acid metabolism	map00220	Arginine biosynthesis	1
Metabolism	Carbohydrate metabolism	map00520	Amino sugar and nucleotide sugar metabolism	4
Metabolism	Carbohydrate metabolism	map00051	Fructose and mannose metabolism	1
Metabolism	Carbohydrate metabolism	map00053	Ascorbate and aldarate metabolism	1
Metabolism	Amino acid metabolism	map00360	Phenylalanine metabolism	1
Metabolism	Amino acid metabolism	map00260	Glycine, serine and threonine metabolism	2
Metabolism	Amino acid metabolism	map00400	Phenylalanine, tyrosine and tryptophan biosynthesis	1

Metabolism	Carbohydrate metabolism	map00030	Pentose phosphate pathway	1
Metabolism	Amino acid metabolism	map00280	Valine, leucine and isoleucine degradation	2
Metabolism	Amino acid metabolism	map00290	Valine, leucine and isoleucine biosynthesis	2
Metabolism	Energy metabolism	map00910	Nitrogen metabolism	1
Metabolism	Carbohydrate metabolism	map00620	Pyruvate metabolism	2
Metabolism	Carbohydrate metabolism	map00500	Starch and sucrose metabolism	1
Genetic Information Processing	Folding, sorting and degradation	map03060	Protein export	1
Genetic Information Processing	Translation	map03008	Ribosome biogenesis in eukaryotes	1
Genetic Information Processing	Folding, sorting and degradation	map04141	Protein processing in endoplasmic reticulum	2
Cellular Processes	Cell growth and death	map04113	Meiosis - yeast	1
Cellular Processes	Transport and catabolism	map04138	Autophagy - yeast	1

KEGG enrichment analysis

Pathway id	Description	Ratio_in_study	Ratio_in_pop	Rich factor	Pvalue
map00040	Pentose and glucuronate interconversions	5/ 66	64/ 4287	0.078125	0.002807
map00520	Amino sugar and nucleotide sugar metabolism	4/ 66	66/ 4287	0.060606	0.018082
map00254	Aflatoxin biosynthesis	1/ 66	3/ 4287	0.333333	0.045489
map00770	Pantothenate and CoA biosynthesis	2/ 66	27/ 4287	0.074074	0.064027
map00350	Tyrosine metabolism	3/ 66	51/ 4287	0.058824	0.042966
map00290	Valine, leucine and isoleucine biosynthesis	2/ 66	19/ 4287	0.105263	0.03373
map00910	Nitrogen metabolism	1/ 66	22/ 4287	0.045455	0.289778
map03060	Protein export	1/ 66	21/ 4287	0.047619	0.278618
map00650	Butanoate metabolism	1/ 66	29/ 4287	0.034483	0.36328
map04141	Protein processing in endoplasmic reticulum	2/ 66	87/ 4287	0.022989	0.389164
map00330	Arginine and proline metabolism	1/ 66	43/ 4287	0.023256	0.48853

map00051	Fructose and mannose metabolism	1/ 66	41/ 4287	0.02439	0.472249
map00630	Glyoxylate and dicarboxylate metabolism	1/ 66	40/ 4287	0.025	0.463918
map00030	Pentose phosphate pathway	1/ 66	27/ 4287	0.037037	0.343081
map00360	Phenylalanine metabolism	1/ 66	27/ 4287	0.037037	0.343081
map01240	Biosynthesis of cofactors	3/ 66	155/ 4287	0.019355	0.429281
map00220	Arginine biosynthesis	1/ 66	20/ 4287	0.05	0.267284
map00400	Phenylalanine, tyrosine and tryptophan biosynthesis	1/ 66	20/ 4287	0.05	0.267284
map00250	Alanine, aspartate and glutamate metabolism	1/ 66	39/ 4287	0.025641	0.455457
map00860	Porphyrin and chlorophyll metabolism	1/ 66	19/ 4287	0.052632	0.255776
map00053	Ascorbate and aldarate metabolism	1/ 66	18/ 4287	0.055556	0.244089
map00620	Pyruvate metabolism	2/ 66	59/ 4287	0.033898	0.230016
map00260	Glycine, serine and threonine metabolism	2/ 66	55/ 4287	0.036364	0.207254
map00270	Cysteine and methionine metabolism	2/ 66	51/ 4287	0.039216	0.184774
map00280	Valine, leucine and isoleucine degradation	2/ 66	47/ 4287	0.042553	0.162704
map04138	Autophagy - yeast	1/ 66	78/ 4287	0.012821	0.705144
map04113	Meiosis - yeast	1/ 66	75/ 4287	0.013333	0.690833
map00500	Starch and sucrose metabolism	1/ 66	71/ 4287	0.014085	0.670684
map03008	Ribosome biogenesis in eukaryotes	1/ 66	71/ 4287	0.014085	0.670684
